# Defining Non–small Cell Lung Cancer Tumor Microenvironment Changes at Primary and Acquired Immune Checkpoint Inhibitor Resistance Using Clinical and Real-World Data

**DOI:** 10.1158/2767-9764.CRC-24-0605

**Published:** 2025-06-30

**Authors:** Lang Ho Lee, Xin Xu, Thanos Mourikis, Fanying Tang, Lauren Fairchild, Lexiang Ji, Angelo L. Grauel, Joel P. Wagner, Sebastian Szpakowski, Marc R. Pelletier, Lisa Kattenhorn, Laurent Sansregret, Carlotta Costa, Claudia Bossen, Heather Burks, Anna F. Farago, Jincheng Wu

**Affiliations:** 1 Novartis Pharmaceuticals Corporation, Cambridge, Massachusetts.; 2 Novartis Pharmaceuticals Corporation, Basel, Switzerland.

## Abstract

Immune checkpoint inhibitors (ICI) have demonstrated clinical efficacy in non–small cell lung cancer (NSCLC), and extensive research has been conducted to explore biomarkers predictive of ICI response. However, the impact of ICI on the tumor and tumor microenvironment at primary and acquired resistance states is understudied due to the difficulty of collecting tissue biopsies at disease progression. In this study, we leveraged clinical and real-world data to study ICI resistance. Data used in this work consist of treatment outcome information and tissue RNA sequencing data from advanced-stage NSCLC cohorts from three sources: the Tempus real-world evidence database; CANOPY-1 (NCT03631199), a phase III clinical trial in first-line NSCLC; and Stand Up To Cancer (SU2C) publication. Our results indicate higher IFNγ and T-cell exhaustion in patients’ tumors at acquired resistance and low levels of B-cell and dendritic cell expression at primary resistance. The lower B-cell and dendritic cell levels may be primarily driven by prior treatment with a platinum-based chemotherapy regimen. Baseline transcriptomics data additionally suggest that innate immune cells may play an antitumor role in PD-L1<1% patients, whereas IFNγ and T-cell inflammation are more predictive of ICI treatment outcomes in PD-L1≥1% patients. Our study suggests a clear divergence of the tumor microenvironment in patients with primary versus acquired resistance and a potential role of myeloid cells in the PD-L1<1% population. These findings shed light on potential next-generation therapies to overcome ICI resistance.

**Significance:**

ICI benefits patients with NSCLC, but resistance remains common. Our research highlights differences in tumor environments between primary and acquired resistance after ICI treatment, emphasizing distinct post-therapy approaches. Findings also suggest myeloid cells as key players in PD-L1–negative cases, guiding future treatment strategies to overcome resistance and improve outcomes.

## Introduction

PD-1 and PD-L1 checkpoint inhibitors have improved clinical outcomes in both early and advanced stages of non–small cell lung cancer (NSCLC; refs. 1–4). However, both primary and acquired resistance are notably prevalent in patients with late-stage NSCLC. Specifically, less than half of the patients exhibit an initial response (5), and among those who do respond initially, a majority still progress because of acquired resistance.

Over the past decade, primary resistance to immune checkpoint inhibitors (ICI) has been extensively researched in clinical studies. Using baseline tissue and cell-free DNA data, various biomarkers have been found to associate with initial response and survival. Factors such as higher PD-L1 levels, T-cell infiltration (6), elevated IFNγ pathway expression, and tumor mutation burden (7) have been reported to be associated with favorable ICI outcomes. In contrast, STK11 mutations (8) and loss of heterozygosity in human leukocyte antigen (HLA; ref. 9) have been linked with worse outcomes. In addition, in patients with EGFR mutations, ICIs did not add significant clinical benefits, potentially due to the lack of tumor neoantigens to trigger antitumor immunity (10).

Characterization of acquired resistance in patients with NSCLC is understudied relative to primary resistance due to difficulties in collecting biopsies at disease progression, and noninvasive biopsies (e.g., cell-free DNA and circulating tumor cells) are hard to infer resistance due to nongenetic mechanisms. Previous studies have relied on limited samples from patients or only preclinical models (5). However, two recent studies provided considerable insight into acquired resistance by comparing paired NSCLC biopsies collected before and after ICI therapy. Memon and colleagues (11) found that increased IFNγ and T-cell exhaustion together with loss of heterozygosity in HLA were the driving factors in acquired resistance. Ricciuti and colleagues (12) reported a lower level of T-cell infiltration after ICI treatments and identified acquired heterozygous loss of genes, including STK11, B2M, and KEAP1.

In this study, we leveraged NSCLC cohorts with multimodal data from the clinical trial CANOPY-1, the academic initiative Stand Up To Cancer (SU2C)–NSCLC, and the Tempus real-world evidence (RWE) database to comprehensively investigate primary and acquired resistance to ICI.

## Materials and Methods

### Tempus NSCLC cohort derivation

Multimodal, deidentified NSCLC data from the Tempus database (Tempus AI, Inc., RRID:SCR_025832) with DNA sequencing, RNA sequencing (RNA-seq), and clinical data were retrospectively analyzed in this study. Tempus patients were separated into three cohorts: (i) cohort 1 (patients with treatment-naïve biopsies), (ii) cohort 2 (patients with biopsies collected after ICI therapy), and (iii) cohort 3 (patients with paired biopsies before and after any treatment). All patients were diagnosed with NSCLC after 2015 and had stage III/IV disease. Additionally, the patients’ tumors had nonsquamous histology, and the patient age was known. In cohorts 1 and 2, patients with actionable *EGFR, BRAF, or MET* mutations or pathogenic fusions involving ALK, *ROS1, NTRK1*, and *NTRK2* were excluded. All data from patients meeting these inclusion/exclusion criteria were licensed from Tempus for cohort 2 (*n* = 326) and cohort 3 (*n* = 56). For cohort 1, 415 patients were randomly selected from the patient pool that met the inclusion/exclusion criteria.

### CANOPY-1 biomarker cohort

The CANOPY-1 study (NCT03631199) was a phase III, randomized, double-blind, global study evaluating the efficacy and safety of pembrolizumab plus platinum-based doublet chemotherapy combined with either canakinumab or placebo as a first-line (1L) therapy with published clinical results (13) and biomarker results (14). Patients with available RNA-seq data and CD8 IHC were included for analysis, and the study was conducted in accordance with the principles of the Declaration of Helsinki and the Good Clinical Practice guidelines of the International Council for Harmonisation. The study protocols and all amendments were reviewed by the independent ethics committee or Institutional Review Board at each center. Patients whose data were used in this study provided written informed consent for additional research. Collection of archival or newly obtained baseline tumor tissue samples during screening was mandatory. PD-L1 expression was assessed using the IHC 22C3 pharmDx assay (13). To assess the level of CD8^+^ immune cells (lymphocytes), a specific duplex chromogenic IHC assay comprising an anti-CD8 antibody (SP239, Abcam, RRID: AB_2756374) and an anti-panCK antibody (AE1/AE3/PCK26, Ventana: Roche Diagnostics, RRID: AB_2941938) was performed on the Ventana DISCOVERY ULTRA (RRID: SCR_021254), and quantitative assessment was carried out using Visiopharm software at CellCarta (RRID: SCR_021711; ref. 15). RNA-seq was generated using RNaseH followed by the TruSeq RNA version 2 Library Preparation kit (Illumina, RRID:SCR_010233). Sequence data were aligned to the reference human genome (build hg19) using STAR version 2.4.0e (RRID: SCR_004463; ref. 16). Mapped reads were then used to quantify transcripts using HTSeq version 0.6.1p1 (RRID: SCR_005514; ref. 17).

### SU2C-NSCLC cohort

For SU2C-NSCLC data (18), a raw gene expression count matrix was curated, and Ensembl IDs were mapped to gene symbols. The raw counts were normalized using the trimmed mean of *M*-value normalization as implemented in the edgeR R package (RRID:SCR_012802; ref. 19).

### Tempus mutation data processing

Tempus genomic data were generated using the Tempus xT CAP/CLIA-validated next-generation sequencing solid tumor assay (20, 21). Tempus xT (version 4) identifies somatic variants [single-nucleotide variants (SNV)], insertions and deletions (indel), and copy-number variants in 648 genes and translocations in 23 genes, depending on version numbers. For the analysis of the association between baseline genetic alterations with clinical outcomes, tissue samples from 297 patients were sequenced using version 4 of the assay; tissue from one patient was sequenced using version 3 of the assay, which detects translocations in 22 genes; and tissue from one patient was sequenced using version 2 of the assay, which profiles SNVs, indels, and copy-number variants in 595 genes, along with translocations in eight genes. Two variant callers, FreeBayes (RRID:SCR_010761) and Pindel (RRID:SCR_000560), were used to identify SNVs and indels in the Tempus data. Paired sample variant calling was performed when normal samples were available. In the absence of normal samples, variants were classified as germline or somatic based on a Bayesian model. The annotation of variants as pathogenic, likely pathogenic, variants of unknown significance, benign, or likely benign was performed using evolutionary, functional, clinical, and known gene–disease relationship information (20). Copy-number estimation was performed using a Tempus-developed copy number algorithm, and fusions were detected using the SpeedSeq analysis pipeline (RRID:SCR_000469; ref. 20), and further filtering was applied to identify structural variants leading to biologically relevant fusion proteins. For short variants (SNVs and short indels), only pathogenic and likely pathogenic variants were considered for downstream analysis, except for short variants in *KEAP1*. All *KEAP1* variants were retained, as this gene is less well annotated.

### Tempus RNA-seq data processing

RNA-seq was conducted using the Tempus xR assay (Tempus AI, Inc.). Briefly, Tempus xR is a whole-exome capture next-generation sequencing assay spanning a 39 Mb target region of the human genome that quantifies transcript- and gene-level expression and identifies transcriptional evidence of chromosomal rearrangements resulting in the expression of fusion RNA species (22).

### Survival analysis

Progression-free survival (PFS) and overall survival (OS) were presented descriptively using the Kaplan–Meier method. Cox proportional hazards (Coxph) regression models were used to investigate the prognostic and predictive roles of biomarkers for OS and to estimate HRs and 95% confidence intervals. For Tempus, OS was inferred as the length of time from the start date of the 1L treatment to the date of death. If there was no evidence of death, patients were censored at the last recorded follow-up date. Real-world PFS (rwPFS) was derived by measuring the duration from the start date of 1L treatment to the date of progression or the date of death. Specifically, progression events were defined when a progression/new metastasis/death event occurred at least 14 days after the treatment start date and before the treatment end date or start date of the subsequent line of treatment. If there was no evidence of progression, patients were censored at either the treatment end date or the subsequent line’s treatment start date if the treatment end date was undefined. Coxph analysis was performed with stage, PD-L1, and KRAS mutation included as covariates. For CANOPY-1, stratification factors [PD-L1 expression and histology (squamous vs. nonsquamous) and geographical regions (East Asia vs. North America and Western Europe vs. the rest of the world)] were all included in the models, consistent with the predefined statistical analysis methods in the study (13), and the treatment arm was also adjusted to model any potential effect from the experimental arm if both arms were included in the Coxph model.

### Downstream unsupervised differential gene expression analysis

limma (RRID:SCR_010943; ref. 23) was used to profile differentially expressed genes. When analyzing the unpaired cohorts, *KRAS* mutation status, PD-L1 level, and biopsy location were included as covariates in the limma model design. For the paired cohort, the differential gene expression analysis was also adjusted by patient ID by using the duplicateCorrelation function in limma to estimate intrapatient variation. Gene expression signatures were curated from MSigDB (RRID:SCR_016863) hallmark (24) and publications (25, 26) listed in Supplementary Table S1. To calculate the gene signature expression score, the mean of normalized gene expression (log scale) was used. The R fgsea package (bioRxiv 060012; RRID:SCR_020938) was used for gene set enrichment analysis using combined hallmark and tumor microenvironment (TME) signature sets as references.

### Downstream supervised analysis

To compare gene signature scores between unpaired cohorts, biopsy location, PD-L1, and *KRAS* mutation status were included as covariates in the lm function, and the estimated marginal means of signatures were calculated using the emmeans package (RRID:SCR_018734; ref. 27). Specifically, the IFNγ pathway was measured by the 18-gene signature (7, 28); immune B-cell and dendritic cell (DC) signatures were used to measure B-cell and DC levels in the TME, respectively (Supplementary Table S1).

### PD-L1 IHC imputation by PD-L1 mRNA

As PD-L1 IHC values were not available for all patients in the Tempus cohort, patients with missing PD-L1 IHC status were imputed by *CD274* mRNA. A total of 482 tissue biopsies had matched PD-L1 IHC (22C3) and RNA-seq. Using this set of training data, the optimal threshold of *CD274* (PD-L1) gene expression was found to predict a PD-L1 score ≥50% by finding the maximum kappa metric at each potential *CD274* expression threshold. When predicting PD-L1 ≥50%, the overall accuracy of the optimal *CD274* expression threshold was 0.84 (sensitivity: 0.79; specificity: 0.88). When predicting PD-L1 ≥1%, the overall accuracy of the optimal *CD274* expression threshold was 0.82 (sensitivity: 0.81; specificity: 0.82). These identified thresholds were used to classify the PD-L1 status of the biopsies with the unknown PD-L1 IHC status (*n* = 393).

### Resistance criteria definition for Tempus real-world data

End of ICI treatment (EOT) samples were defined as those biopsies collected within 30 days before or after the EOT or ICI + chemotherapy treatment. If the treatments’ end date information was missing, EOT samples were defined as biopsies collected at least 60 days after the ICI or ICI + chemotherapy treatment start date. Among the EOT samples, resistance samples were defined as those whose corresponding next line of treatment started within 90 days of EOT or if a progressive disease event was recorded within 90 days of the sample collection date. Progressive disease events were defined as metastasis (second malignancy), disease progression, and death. Resistance samples were further split into primary resistance (ICI treatment duration of less than 180 days) and acquired resistance (ICI treatment duration of greater than or equal to 180 days).

### NSCLC single-cell RNA-seq data processing

A publicly available lung cancer single-cell RNA-seq dataset (29) was downloaded and processed. This dataset consisted of single-cell RNA-seq data aggregated from 29 datasets, containing 556 samples and 1.28 million single cells. The expression count matrix from this publication was processed using Seurat (30), and cell type annotation from the publication was used directly. To calculate gene signature scores by cell type, single-cell expression was first converted into pseudobulk expression, in which gene expression data from groups of cells within the same cell type from the same patient were aggregated. The mean of the log-scale trimmed mean of *M*-value normalized count per million values was calculated using edgeR for all the genes in the signature for a given sample to form the signature score.

### Data availability

All data associated with this study are present in the article or the Supplementary Materials, except for patient-level data for the CANOPY-1 and Tempus cohorts. The CANOPY-1 trial identification and treatments administered cannot be reported on a per-individual basis to preserve patient anonymity. Processed data and clinical information for patients will be made available upon reasonable request. For Tempus cohorts, the patient-level data are not publicly available due to the license terms. Tempus may make access to data available pending a signed data use agreement. Requests can be sent to publication.inquiry@tempus.com. For SU2C-NSCLC, the patient-level data are available via the original publication (18) as specified in the Materials and Methods section.

## Results

### Cohort overview

Data studied in this work consist of five cohorts of advanced-stage NSCLC from three sources: (i) three cohorts licensed from the Tempus RWE database; (ii) one cohort from CANOPY-1 (NCT03631199), a phase III clinical trial in 1L NSCLC (13); and (iii) one cohort curated from the academic initiative SU2C (18). The cohort size, stage, treatment history, and histology subtype are summarized in Table 1.

**Table 1:** Key patient characteristics of the five cohorts in the study

Cohort	Prior treatment	Stage	Checkpoint regimen	Histology	Number of patients with RNA-seq
Tempus cohort 1	Treatment-naïve	III/IV	PD-L1 ± chemo	Nonsquamous	415
Tempus cohort 2	ICI ± chemotherapy	III/IV	PD-L1 ± chemo	Nonsquamous	326
Tempus cohort 3	Mixed (naïve, ICI, chemo, and tyrosine kinase inhibitor)	III/IV	Mixed	Nonsquamous	56
CANOPY-1	Treatment-naïve	IIIB/IV	PD-L1 ± chemo ± canakinumab	Squamous and nonsquamous	298
SU2C-NSCLC	Mixed (naïve, ICI, chemo, and tyrosine kinase inhibitor)	III/IV	PD-L1 ± CTLA4	Squamous and nonsquamous	153

In brief, three cohorts from Tempus were licensed from Tempus in April 2023 and included a treatment-naïve biopsy cohort (Tempus cohort 1, *n* = 415), a cohort with biopsies collected after ICI ± chemotherapy (Tempus cohort 2, *n* = 326), and a cohort with paired biopsies (Tempus cohort 3, *n* = 56; Supplementary Table S2). Specifically, patients in cohort 3 have two or more tissue biopsies with at least one cancer treatment regimen between the two biopsies (see “Materials and Methods” for more details).

The CANOPY-1 cohort contained 298 patients with RNA-seq data generated from baseline tissue biopsies. Patients were diagnosed with locally advanced or metastatic NSCLC and were treated with pembrolizumab plus platinum-based doublet chemotherapy combined with either canakinumab or placebo as their first-line therapy. SU2C-NSCLC cohort data were curated from the original publication (18) and contained 153 patients treated with a PD-1 or PD-L1 inhibitor as monotherapy or in combination with a CTLA-4 inhibitor in the first-line/second-line (2L) setting. All biopsies in the SU2C cohort were collected prior to ICI treatment.

### Baseline innate immune cells are associated with better ICI outcomes in PD-L1 <1% patients

**Figure 1:**
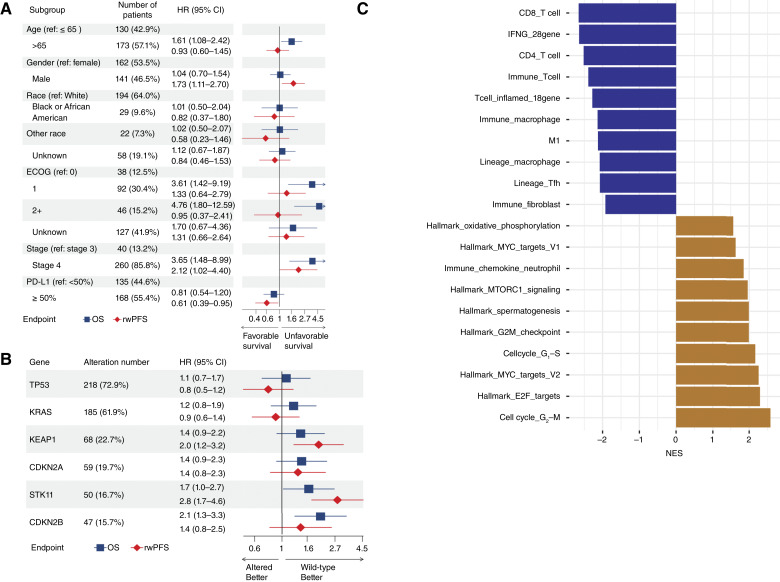
Baseline demographic and transcriptomic features associated with clinical outcomes in Tempus cohort 1. **A,** Forest plot of baseline demographic features in association with clinical outcomes (OS and rwPFS) in 1L NSCLC ICI treatment. **B,** Forest plot of the most frequently observed baseline genomic alterations in association with 1L ICI treatment outcomes (OS and rwPFS). HRs >1 indicate worse outcomes for patients whose tumors harbor a genomic alteration relative to patients with wild-type tumors. **C,** Top enriched pathways and signatures associated with ICI OS using baseline RNA-seq data. Negative normalized enrichment scale (NES) was associated with longer OS. CI, confidence interval; ECOG, Eastern Cooperative Oncology Group; ref, reference.

We first examined the baseline demographic, transcriptomic, and genomic features associated with ICI treatment outcomes in Tempus cohort 1, the treatment-naïve cohort. Among 415 patients, 299 received ICI ± chemotherapy as their 1L treatments with complete line-of-therapy information (i.e., complete regimen information and treatment start date). Among baseline demographic and diagnostic features, stage IV versus III showed an association with shorter rwPFS and OS, whereas PD-L1 ≥50% showed an association with longer rwPFS and OS compared with PD-L1 <50% (Fig. 1A). Among genomic alterations, *STK11*, *KEAP1* loss-of-function alterations, and *CDKN2B* deep deletions showed an association with shorter rwPFS and OS (Fig. 1B). In an unbiased transcriptomic gene set enrichment analysis, T-cell and IFNγ pathways were associated with better outcomes, whereas the cell cycle, c-Myc pathway, and neutrophil-recruiting chemokines were associated with worse outcomes (Fig. 1C). Overall, these findings were consistent with known prognostic and predictive biomarkers for PD-1 and PD-L1 inhibitors in CANOPY-1 (14) and SU2C-NSCLC studies (18).

**Figure 2:**
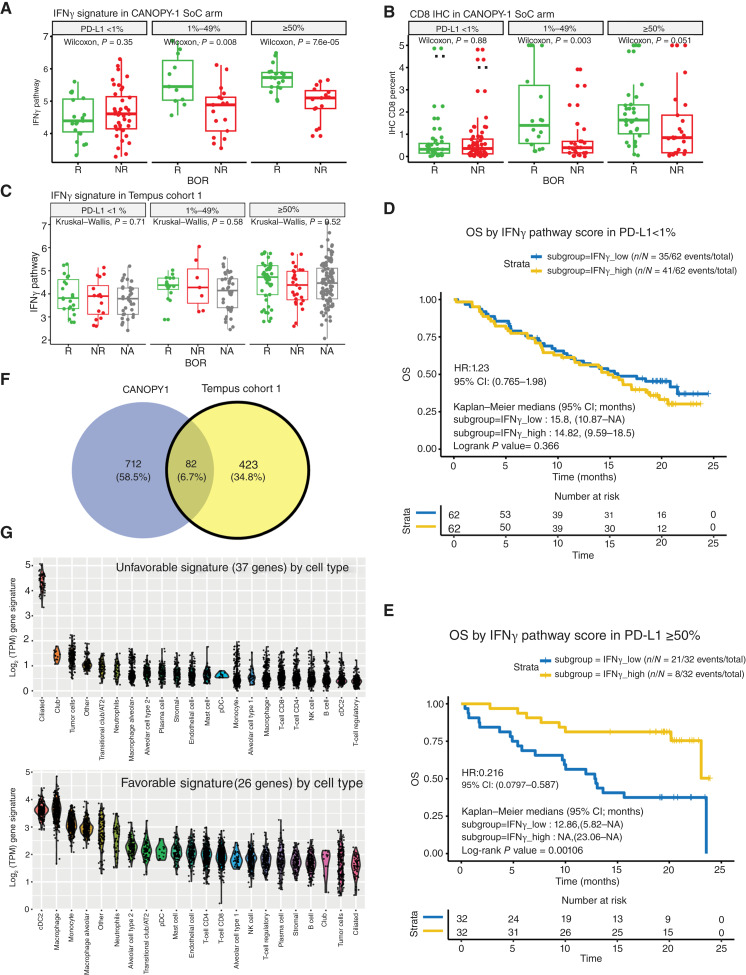
Baseline gene expression associated with clinical outcomes in patients with PD-L1 <1% utilizing data from both Tempus cohort 1 and CANOPY-1 studies. **A,** Baseline IFNg expression scores measured by RNA-seq in responders (R) and nonresponders (NR) in the CANOPY-1 study’s Standard-of-Care (SoC) arm (pembrolizumab ± chemotherapy). **B,** Baseline CD8 IHC percentages in the carcinoma region in responders and nonresponders in the CANOPY-1 study’s SoC arm; BOR in CANOPY-1 was assessed by RECIST 1.1 criteria. **C,** Baseline IFNγ expression scores measured by RNA-seq in the Tempus ICI cohort in responders and nonresponders as defined by Tempus according to real-world clinical note interpretation. **D** and **E,** OS of patients with high and low IFNg pathway scores in the (**D**) PD-L1 <1% subgroup and (**E**) PD-L1 ≥50% subgroup. **F,** Venn diagram of genes with significant association (*P* value < 0.05) with OS for patients in the PD-L1 <1% subgroup in both the CANOPY-1 and Tempus cohorts. **G,** Expression level of unfavorable (top) and favorable gene expression signatures (bottom) in NSCLC single-cell RNA-seq data categorized by cell type. Each point in the violin represents the signature score for the given cell type from one patient sample. CI, confidence interval.

As PD-L1 IHC is a known biomarker for ICI treatment outcomes, we further explored whether the IFNγ pathway showed a consistent association with outcomes across PD-L1 subgroups using the CANOPY-1 cohort and Tempus cohort 1. The SU2C cohort was not included in this analysis because of a high rate of missing PD-L1 IHC results. Interestingly, in CANOPY-1, baseline IFNγ was higher in responders than in nonresponders in the PD-L1–positive subgroups (PD-L1 1%–49% and PD-L1 ≥50%), but this association was weaker or not observed in the PD-L1 <1% subgroup (Fig. 2A). Similar results were found in CD8 IHC data (Fig. 2B). In Tempus, the baseline IFNγ score did not differ statistically between responders and nonresponders in all PD-L1 subgroups (Fig. 2C), but a numerical trend was observed in the PD-L1 ≥50% subgroup (Kruskal–Wallis *P* value = 0.52). Compared with RECIST-based response criteria, Tempus RWE data used clinical notes to determine response categories during the line of therapy. As a result, some patients had missing or unknown best overall response (BOR) information, which may have reduced the ability to identify differences between the BOR groups. Nonetheless, the difference between the PD-L1 <1% and PD-L1 ≥50% subgroups was validated through OS outcomes in CANOPY-1 (Fig. 2D and 2E), in which patients with higher IFNγ scores saw longer OS only in the PD-L1 ≥50% subgroup but no difference in the PD-L1 <1% subgroup. A similar trend was also observed in Tempus OS that IFNγ scores showed a trend of favorable OS outcomes only in PD-L1 high subgroups (HR = 0.63, *P* value = 0.09 for PD-L1 ≥50% vs. HR = 1.11, *P* value = 0.79 for PD-L1 <1%; Supplementary Fig. S1). Two hypotheses may explain this observation: (i) The initial response was driven by chemotherapy in PD-L1 <1% patients and (ii) biomarkers other than IFNγ were associated with checkpoint treatment.

To examine these hypotheses, an unbiased analysis was conducted to individually screen the expression of all genes in PD-L1 <1% patients from both the CANOPY-1 cohort and the Tempus cohort 1 using OS as the endpoint (Supplementary Table S3). A total of 82 genes were significantly associated with OS in both cohorts (Fig. 2F; Supplementary Table S4). To understand the source of expression by cell type, the gene set was projected onto single-cell NSCLC RNA-seq datasets (29). Of the 82 genes, 63 were detectable in the single-cell atlas. Among these 63 genes, 26 were associated with longer OS and were highly expressed in innate immune cells, including DCs, macrophages, and monocytes, whereas 37 genes were associated with shorter OS and were highly expressed in ciliated, club, and tumor cells (Fig. 2G). These results indicate that innate immune cells may play a role in antitumor immunity in patients with PD-L1–negative NSCLC. In contrast, although ciliated cells are reported to be able to increase risk of lung adenocarcinoma (31), there is little research on this cell type in lung cancer setting. In addition to these results from single-cell data, these unfavorable genes were also enriched in genes associated with bronchial epithelial cell differentiation to the airway epithelium (32). Given that it is hard to assess from bulk RNA-seq data whether this gene expression pattern is specific to malignant cells or nonmalignant ciliated cells, the significance of this finding is unclear.

### Higher IFNγ and T-cell exhaustion are observed in acquired resistance to ICI treatment

In addition to the characterization of baseline molecular features, understanding tumor and TME changes at disease progression is critical to understanding and potentially overcoming treatment resistance, especially acquired resistance. In this section, we focused on using the three Tempus cohorts to study resistance at the post-ICI state using both unpaired and paired cohorts.

The unpaired cohort analysis utilized Tempus cohort 1 (pre-ICI, *n* = 415) and Tempus cohort 2 (post-ICI, *n* = 326). Additionally, post-ICI biopsies from Tempus cohort 2 were categorized into primary and acquired resistance groups (Supplementary Fig. S2). Specifically, to classify patients as having primary or acquired resistance, they must have met the following criteria: (i) Patients had a documented ICI treatment start and end date, with a biopsy collected within a 30-day window after the ICI treatment ended (*n* = 301 out of 326), and (ii) within 90 days following the EOT, patients either moved to a new line of therapy or had a disease progression assessment (*n* = 134 out of 301). Of the patients who met these two criteria, those who remained on ICI treatment for longer than 180 days were classified as having acquired resistance (*n* = 75 out of 134). Those with shorter treatment durations were categorized as having primary resistance (*n* = 59 out of 134).

**Figure 3:**
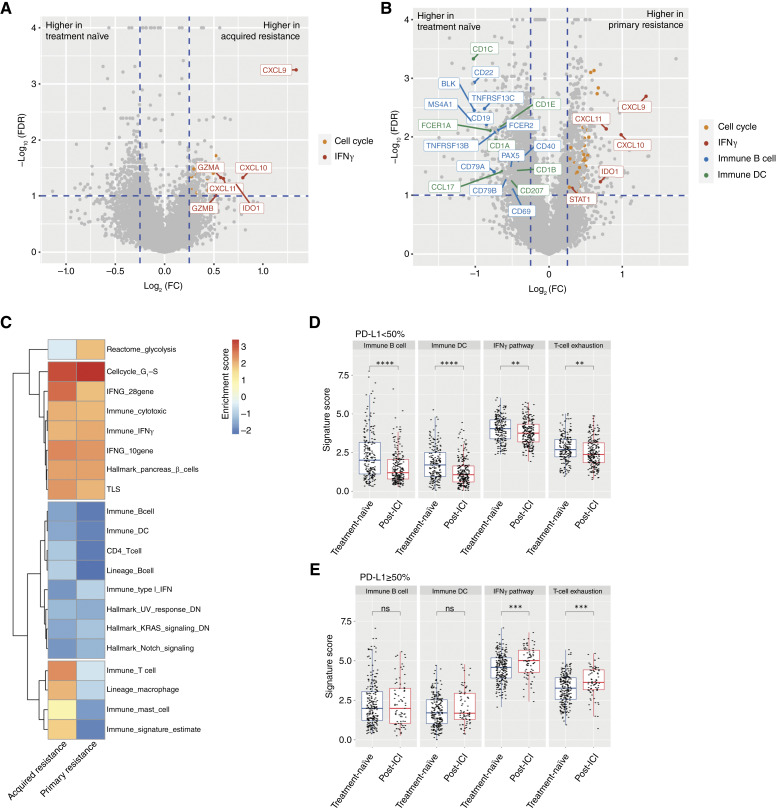
Analysis of the TME between post-ICI cohort (Tempus cohort 2) and treatment-naïve cohort (Tempus cohort 1). **A,** Volcano plot of differential expression analysis comparing the post-ICI acquired resistance subgroup vs. treatment-naïve cohort. **B,** Volcano plot of differential expression analysis comparing the post-ICI primary resistance subgroup vs. treatment-naïve cohort. **C,** Gene set enrichment analysis results of hallmark pathways and TME signatures with significant associations in either primary or acquired resistance cohorts. A positive normalized enrichment scale score indicates the increased pathway/signature in resistance cohorts relative to the treatment-naïve cohort. **D** and **E,** Box plots of B cell, DC, IFNγ, and T-cell exhaustion signatures for the (**D**) PD-L1 <50% subgroup and (**E**) PD-L1 ≥50% subgroup in post-ICI and treatment-naïve samples. FC, fold change; NES, normalized enrichment scale.

By comparing the acquired resistance and treatment-naïve cohorts, we observed both similarity and divergence of TME changes between primary resistance and acquired resistance in the post-ICI setting. Specifically, IFNγ pathway genes were among the most upregulated genes in both primary and acquired resistance, including *CXCL9/10/11, IDO1, GZMA*, and *GZMB* (Fig. 3A and B). In addition, clear downregulation of B-cell and DC genes was observed only in primary resistance (e.g., *CD19, CD22*, and *CD79A* for B cells and *CD1A/CD1B/CD1C/CD1E* for DCs). Enrichment analysis using HALLMARK and TME signatures showed similar trends for B-cell, DC, and IFNγ signatures in primary and acquired resistance (Fig. 3C).

Given that bulk gene expression could be strongly affected by biopsy tissue location, we further investigated whether biopsy tissue location may confound this finding. Indeed, more biopsies were collected from metastatic lesions (lymph node, liver, brain, etc.) in the post-ICI cohorts relative to the treatment-naïve cohort (Supplementary Fig. S3). As a result, we further stratified this comparison by biopsy location (lung, lymph node, liver, and others; Supplementary Fig. S4A) and by PD-L1 subgroups (Fig. 3D). The decrease in B-cell and DC gene expression was observed across all biopsy locations except the liver in the PD-L1 <50% subgroup (Supplementary Fig. S4A). Changes in biopsies collected from liver sites were not conclusive, given a relatively small sample size and lower baseline immune levels. Also, as expected, the decrease in B-cell and DC genes was more significant in the PD-L1 <1% subgroup than in the PD-L1 1% to 49% subgroup (Supplementary Fig. S5). In contrast, the increase in IFNγ pathway expression was more pronounced in biopsies collected from lymph nodes in the PD-L1≥ 50% subgroup, and this increase was in concordance with higher T-cell exhaustion signatures (Fig. 3E; Supplementary Fig. S4B).

In Tempus cohort 3 (paired biopsies cohort), the PD-L1 < 50% subgroup showed a consistent decrease in B-cell and DC signatures but no increase in IFNγ or T-cell exhaustion (Supplementary Fig. S6). However, paired analysis was limited by having only 12 patients who met the criteria for post-ICI resistance (*n* = 6 for primary resistance and *n* = 6 for acquired resistance). Also, the majority of these pairs had biopsies from mismatched tissue locations. These limitations make it hard to reliably interpret the results in the paired cohort analysis.

### Platinum-based therapy leads to colder TME

**Figure 4:**
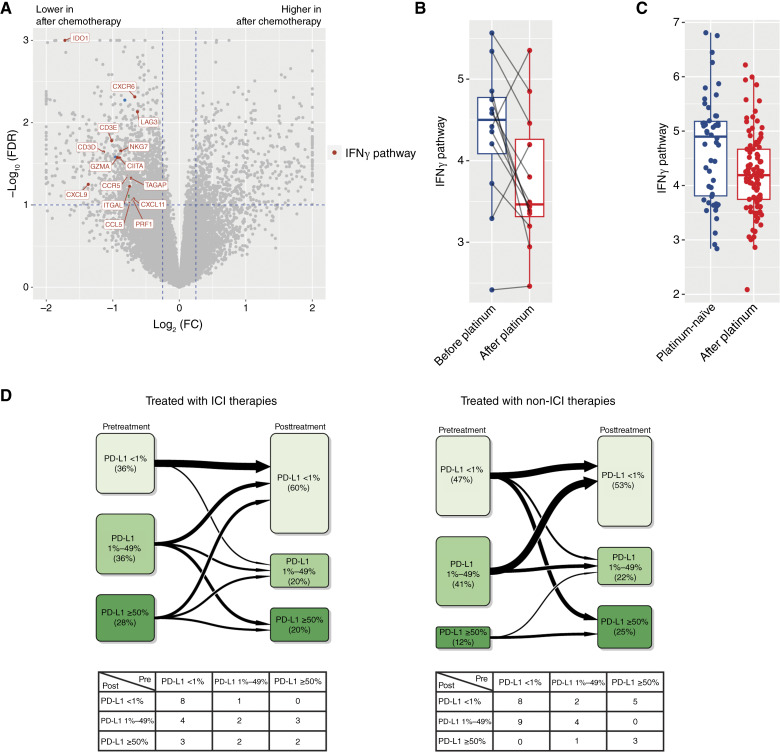
Analysis of the TME after monochemotherapy in the Tempus cohort 3 and SU2C-NSCLC cohorts. **A,** Volcano plot of differential expression analysis for 12 patients treated with platinum-based chemotherapy within Tempus cohort 3. **B,** Box plot of IFNγ signature expression before and after platinum therapy in 12 patients with paired biopsies. **C,** Box plot of the IFNγ signature by prior platinum-based therapy history in the SU2C-NSCLC cohort. **D,** Schematic of PD-L1 IHC class switching observed in Tempus cohort 3 by treatment class (ICI vs. non-ICI).

Given that the majority of NSCLC first-line patients are treated with ICI in combination with chemotherapy, we further explored whether chemotherapy regimens influence IFNγ, B-cell, and DC gene expression changes using the 12 patients from the Tempus cohort 3 treated with platinum-based monotherapy. In these patient samples, IFNγ pathway expression decreased after treatment (Fig. 4A and B). Similarly, this decrease in IFNγ was also observed in SU2C-NSCLC in the post-platinum cohort relative to the platinum-naïve cohort (Fig. 4C). In addition to gene expression changes, changes in PD-L1 status were also investigated in the paired cohort. Of 25 patients, 13 experienced a switch in PD-L1 IHC class after ICI treatment, and there was a mild trend toward PD-L1 decrease. A similar trend was observed in non-ICI–treated patients (17/32 switched class; Fig. 4D). Both ICI and non-ICI treatment groups showed a slight decrease in PD-L1 levels (Supplementary Fig. S7). Taken together, platinum-based chemotherapy may result in a colder TME during disease progression, and the PD-L1 class switch was observed independent of teratment type (ICI vs. chemo).

## Discussion

ICI therapy has shown lasting benefits for patients with NSCLC who initially respond to treatment. It is well known that IFNγ activity is associated with PD-L1 expression (33, 34), and baseline immune infiltrations (T cells, B cells, and DCs) are associated with better ICI outcomes (35, 36). Further investigation of the mechanisms behind the eventual progression in these initial responders could provide valuable insights for developing new therapies to combat this resistance.

Our study indicated a clear divergence of the TME in patients with primary resistance versus acquired resistance. Specifically, tumors of patients experiencing acquired resistance continue to have high IFNγ and T-cell infiltration, whereas tumors from patients experiencing primary resistance show a decrease in B-cell and DC signatures. This divergence may justify the development of different treatment approaches in the post-ICI 2L+ setting for NSCLC. Molecules that could engage and reinvigorate exhausted T cells may better serve acquired resistance patients, whereas molecules with less dependency on immune cells, such as antibody–drug conjugates or radioligand therapies, may fit better for primary resistance patients.

In addition, understanding tumor antigen expression in the post-ICI setting is crucial for next-generation therapy. Novel targets such as TROP2 and CEACAM-5 were tested in the 2L setting; the unbiased gene expression comparison between naïve and post-ICI confirmed that the expressions of both targets were maintained at 2L, and more generally, the transcriptome comparison performed in our study could be leveraged to better position future emerging targets. The full transcriptome comparison is available in Supplementary Tables S5 and S6 and could complement what we have already learned from early-stage/treatment-naïve patients (26, 37, 38).

Due to the multiple clinical cohorts included in our analysis, RNA-seq and PD-L1 IHC data were generated using different assays, and PD-L1 status information was frequently absent in the SU2C cohort. Therefore, the focus of our baseline meta-analysis was on the genes associated with clinical outcomes across studies, whereas study- or cohort-specific analyses were not interpreted because of the difficulty to distinguish technical causes from clinical/biological causes. The finding that myeloid cells (DCs, macrophages, and monocytes) showed stronger prediction of ICI outcomes in PD-L1–negative tumors could be an area for further research. Recent preclinical research (39, 40) has shown that PD-L1 blockade could increase macrophage phagocytosis to reduce tumor growth in mouse models. However, molecules aimed at reducing myeloid immunosuppression in solid tumors have not yet proven to be efficacious in clinical studies. Leveraging myeloid cells in the NSCLC TME is both a challenge and an opportunity in the PD-L1–negative and/or ICI-refractory population.

Compared with two other recent studies (11, 12), our findings confirmed an increase in both IFNγ and T-cell exhaustion in acquired resistance patients (11). However, Ricciuti and colleagues (12) reported that overall T-cell infiltration decreased with increased distance between the location of the T cells and the tumor region. Differences in assay types could potentially explain this discrepancy: image-based IHC/FIHC could measure spatial information and T-cell infiltration quantified as a percentage of cells, whereas bulk RNA-seq focuses on the functional IFNγ genes more quantitatively from the whole biopsy slide, including the stroma region. High-resolution assays such as spatial transcriptomics or single-cell RNA-seq may be better able to examine the position and functional state of T cells. In addition, the expression level of HLA genes was found to be lower in acquired resistance patients, consistent with both studies. In contrast to the two reports, the increased incidence of B2M mutation in acquired resistance patients was not reaffirmed in our analysis. Finally, Memon and colleagues (11) showed a similar trend of decreased B cells and DC cells in acquired and primary resistance cohorts.

Our work is limited by the size of our paired biopsy dataset. Although the Tempus paired cohort has 56 patients, only a few could be used for analysis due to the patients’ resistance status being unclear and mismatched biopsy locations before and after treatment. Therefore, we considered the unpaired analysis to be more reliable as it had a bigger sample size and greater ability to adjust for potential confounding factors (biopsy location, PD-L1 level, etc.).

## Supplementary Material

Supplementary Figure S1 Forest plot of association of baseline IFNg signature with overall survival by each PD-L1 subgroup in Tempus and CANOPY-1 cohorts.

Supplementary Figure S2 Criteria to define post-ICI patients (n = 326) into acquired (n = 75) and primary resistance (n = 59) in Tempus cohort

Supplementary Figure S3 Distribution of biopsy tissue sites comparing the treatment-naïve group (cohort 1) with the post-ICI group (cohort 2) in the left two panels and contrasting primary resistance with acquired resistance within cohort 2 in the right two panels

Supplementary Figure S4 Immune signature expression (B cell, DC, IFNg pathway and T cell exhaustion) by biopsy location and PD-L1 subgroup in unpaired treatment naïve and post-ICI samples

Supplementary Figure S5 Immune signature expression (B cell, DC, IFNg pathway and T cell exhaustion) in PD-L1 <1% and PD-L1 1% to 49% treatment naïve and post-ICI samples

Supplementary Figure S6 Analysis of TME between pre- and post-ICI samples in patients with paired biopsies (Tempus cohort 3).

Supplementary Figure S7 Expression changes of PD-L1 mRNA (CD274) pre- and post-ICI treatment for paired biopsies, grouped by ICI therapy (left panel), and non-ICI therapy (right panel)

Supplementary Table S1 List of gene expression signatured

Supplementary Table S2 Patient characteristics of three Tempus cohorts for age, gender, race and PD-L1 class

Supplementary Table S3 Full table of baseline gene expression association with ICI treatment outcome (OS) in PD-L1 negative in both CANOPY-1 and Tempus cohort 1 patients

Supplementary Table S4 Baseline gene expression with significant association with ICI treatment outcome (OS) in PD-L1 negative in both CANOPY-1 and Tempus cohort 1 patients

Supplementary Table S5 Table of differentially expressed gene table in primary resistance compared to treatment naïve cohort

Supplementary Table S6 Table of differentially expressed gene table in acquired resistance compared to treatment naïve cohort
