# Supplementary material for: Defining Non–small Cell Lung Cancer Tumor Microenvironment Changes at Primary and Acquired Immune Checkpoint Inhibitor Resistance Using Clinical and Real-World Data
Source: Cancer Res Commun. 2025 Jun 30;5(6):1049–59. doi: 10.1158/2767-9764.CRC-24-0605 (PMC12207206; doi:10.1158/2767-9764.CRC-24-0605)

**Supplementary Figure S1. Forest plot of association of baseline IFNγ signature with overall survival by each PD-L1 subgroup in Tempus and CANOPY-1 cohorts.**

| Study | Subgroup | Number of patients | HR.(95%.CI) |
|---|---|---|---|
| CANOPY−1 | PD−L1 < 1% | 124 | 1.23 (0.77, 1.98) |
| | PD−L1 1−49% | 69 | 1.39 (0.63, 3.05) |
| | PD−L1 >= 50% | 64 | 0.22 (0.08, 0.59) |
| Tempus c1 | PD−L1 < 1% | 72 | 1.11 (0.50, 2.49) |
| | PD−L1 1−49% | 58 | 0.67 (0.28, 1.58) |
| | PD−L1 >= 50% | 164 | 0.63 (0.36, 1.09) |

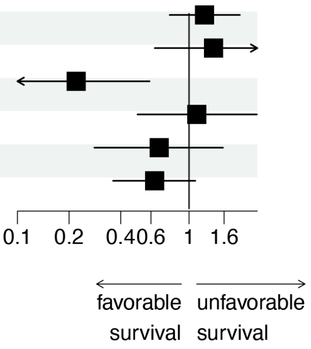

Supplement: Supplementary Figure S1 — Forest plot of association of baseline IFNg signature with overall survival by each PD-L1 subgroup in Tempus and CANOPY-1 cohorts. [file crc-24-0605_supplementary_figure_s1_suppsf1.pdf]
