# Supplementary material for: Defining Non–small Cell Lung Cancer Tumor Microenvironment Changes at Primary and Acquired Immune Checkpoint Inhibitor Resistance Using Clinical and Real-World Data
Source: Cancer Res Commun. 2025 Jun 30;5(6):1049–59. doi: 10.1158/2767-9764.CRC-24-0605 (PMC12207206; doi:10.1158/2767-9764.CRC-24-0605)

Supplementary Figure S2. Criteria to define post-ICI patients (n = 326) into acquired (n=75) and primary resistance (n=59) in Tempus cohort.

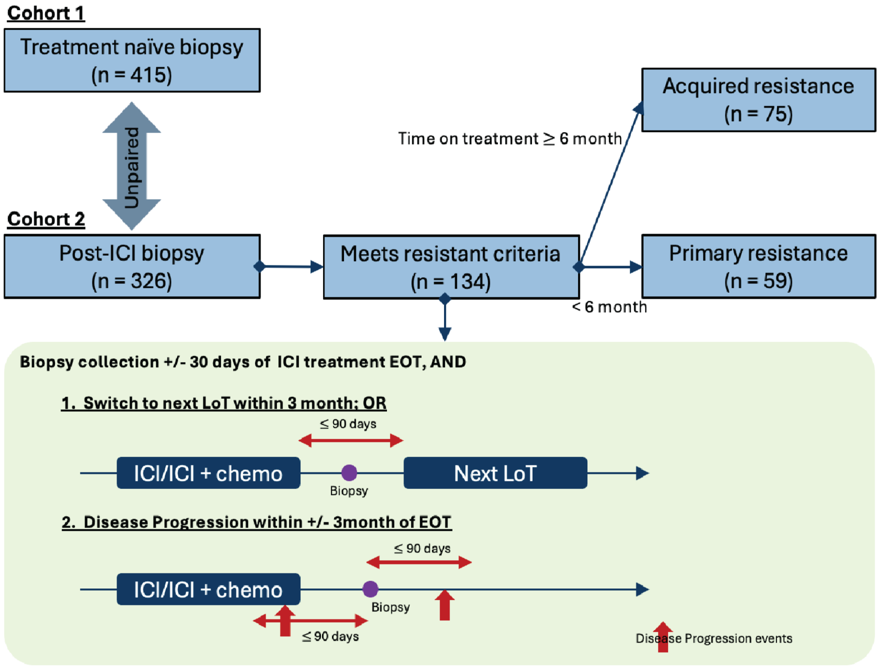

Supplement: Supplementary Figure S2 — Criteria to define post-ICI patients (n = 326) into acquired (n = 75) and primary resistance (n = 59) in Tempus cohort [file crc-24-0605_supplementary_figure_s2_suppsf2.pdf]
