# Supplementary material for: Defining Non–small Cell Lung Cancer Tumor Microenvironment Changes at Primary and Acquired Immune Checkpoint Inhibitor Resistance Using Clinical and Real-World Data
Source: Cancer Res Commun. 2025 Jun 30;5(6):1049–59. doi: 10.1158/2767-9764.CRC-24-0605 (PMC12207206; doi:10.1158/2767-9764.CRC-24-0605)

Supplementary Figure S3. Distribution of biopsy tissue sites comparing the treatment-naïve group (cohort 1) with the post-ICI group (cohort 2) in the left two panels and contrasting primary resistance with acquired resistance within cohort 2 in the right two panels. The lung, lymph nodes, brain, and liver were the most sampled biopsy locations, while "Others" refers to sites either ranked below the top seven categories or were unknown.

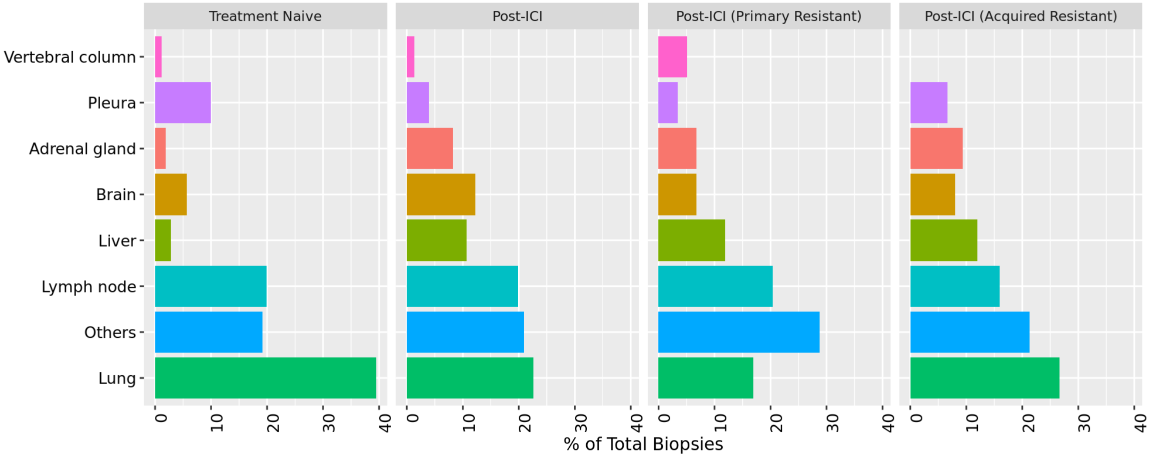

Supplement: Supplementary Figure S3 — Distribution of biopsy tissue sites comparing the treatment-naïve group (cohort 1) with the post-ICI group (cohort 2) in the left two panels and contrasting primary resistance with acquired resistance within cohort 2 in the right two panels [file crc-24-0605_supplementary_figure_s3_suppsf3.pdf]
