# Supplementary material for: Defining Non–small Cell Lung Cancer Tumor Microenvironment Changes at Primary and Acquired Immune Checkpoint Inhibitor Resistance Using Clinical and Real-World Data
Source: Cancer Res Commun. 2025 Jun 30;5(6):1049–59. doi: 10.1158/2767-9764.CRC-24-0605 (PMC12207206; doi:10.1158/2767-9764.CRC-24-0605)

Supplementary Figure S4. Immune signature expression (B cell, DC, IFNγ pathway and T cell exhaustion) by biopsy location and PD-L1 subgroup in unpaired treatment naïve and post-ICI samples. Unpaired t-test was used for each comparison, * $p < 0.05$, ** $p < 0.01$, *** $p < 0.001$, **** $p < 0.0001$

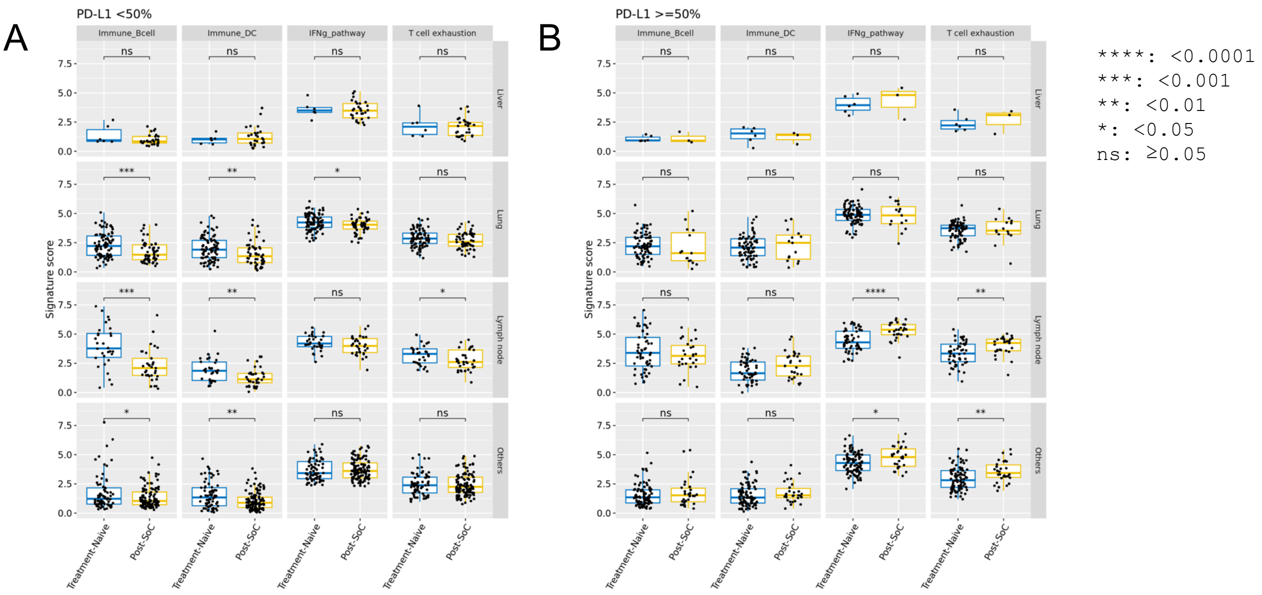

Supplement: Supplementary Figure S4 — Immune signature expression (B cell, DC, IFNg pathway and T cell exhaustion) by biopsy location and PD-L1 subgroup in unpaired treatment naïve and post-ICI samples [file crc-24-0605_supplementary_figure_s4_suppsf4.pdf]
