# Supplementary material for: Defining Non–small Cell Lung Cancer Tumor Microenvironment Changes at Primary and Acquired Immune Checkpoint Inhibitor Resistance Using Clinical and Real-World Data
Source: Cancer Res Commun. 2025 Jun 30;5(6):1049–59. doi: 10.1158/2767-9764.CRC-24-0605 (PMC12207206; doi:10.1158/2767-9764.CRC-24-0605)

Supplementary Figure S6. Analysis of TME between pre- and post-ICI samples in patients with paired biopsies (Tempus cohort 3). (A) Cohort details for patients with paired treatment naive and post treatment biopsies. Of 56 patients with paired biopsies, 25 received ICI treatment(s) between biopsy collection dates, 12 received treatment with only chemotherapy, and 19 received treatment with TKI or other therapy class; (B) Correlation of the change in expression of HALLMARK and TME gene signatures between unpaired and paired cohorts in PD-L1 <50% (left) and PD-L1 >= 50% (right). (C) Boxplot of B cell, DC, IFNγ and T cell exhaustion signatures for PD-L1 < 50% and PD-L1 >= 50% subgroups. Colors of the points indicate the biopsy site locations (lung, lymph node, liver and others/unknown).

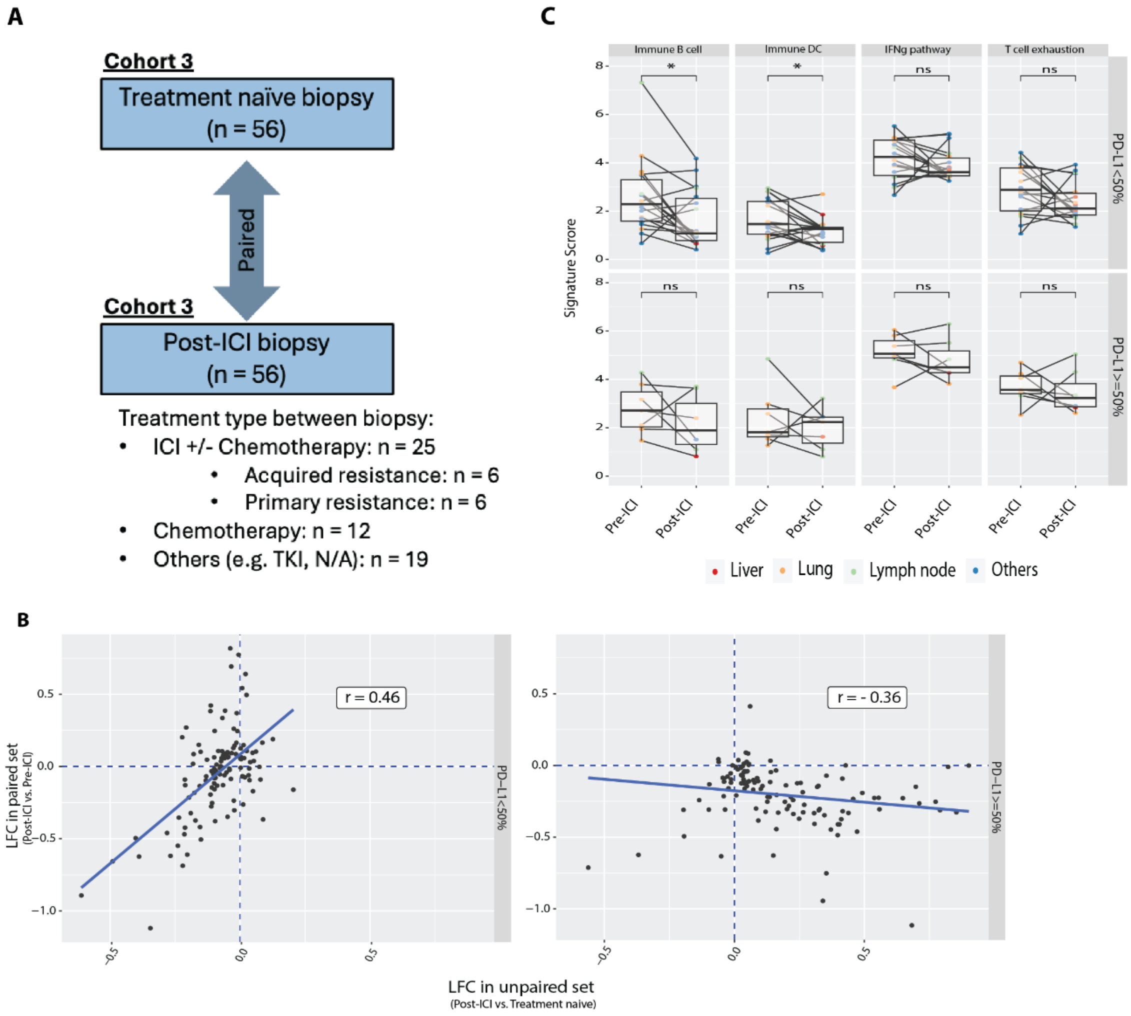

Supplement: Supplementary Figure S6 — Analysis of TME between pre- and post-ICI samples in patients with paired biopsies (Tempus cohort 3). [file crc-24-0605_supplementary_figure_s6_suppsf6.pdf]
