# Supplementary material for: Defining Non–small Cell Lung Cancer Tumor Microenvironment Changes at Primary and Acquired Immune Checkpoint Inhibitor Resistance Using Clinical and Real-World Data
Source: Cancer Res Commun. 2025 Jun 30;5(6):1049–59. doi: 10.1158/2767-9764.CRC-24-0605 (PMC12207206; doi:10.1158/2767-9764.CRC-24-0605)

Supplementary Figure S7. Expression changes of PD-L1 mRNA (CD274) pre- and post-ICI treatment for paired biopsies, grouped by ICI therapy (left panel), and non-ICI therapy (right panel).

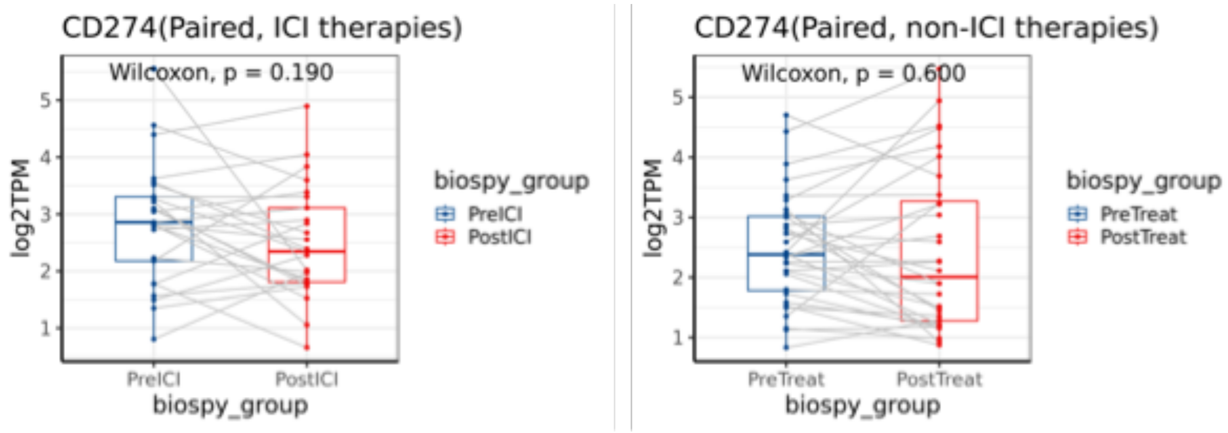

Supplement: Supplementary Figure S7 — Expression changes of PD-L1 mRNA (CD274) pre- and post-ICI treatment for paired biopsies, grouped by ICI therapy (left panel), and non-ICI therapy (right panel) [file crc-24-0605_supplementary_figure_s7_suppsf7.pdf]
