# Supplementary material for: Defining Non–small Cell Lung Cancer Tumor Microenvironment Changes at Primary and Acquired Immune Checkpoint Inhibitor Resistance Using Clinical and Real-World Data
Source: Cancer Res Commun. 2025 Jun 30;5(6):1049–59. doi: 10.1158/2767-9764.CRC-24-0605 (PMC12207206; doi:10.1158/2767-9764.CRC-24-0605)
Supplement: Supplementary Table S1 — List of gene expression signatured [file crc-24-0605_supplementary_table_s1_suppst1.pdf]

Supplementary Table S1. List of gene expression signatured used in the analysis

Signature name	Source	gene
**CAF_ShirleyLiu**	(*1*)	COL3A1,CRISPLD2,COL5A1,COL5A2,COL6A1,COL6A3,CDR1,COL8A1,FBLN1,THBS2,SPARC,COL12A1,FBN1,MXRA5,EFEMP1,SFRP2,THY1,RARRES2,IGFBP5,COL1A2,CCN1,TIMP1,AEBP1,TIMP2,ADAMDEC1,SULF1,C11orf96,TMEM158,VCAN,SERPINF1,CTHRC1,C1R,C1S,CXCL14,C3,PLAT,ISLR,MMP1,GREM1,MMP3,LUM,CHI3L1,DCN,CXCL1,CTSK,DKK3,BGN,COL1A1,PCOLCE
**CD4_Tcell_AvivRegev**	(*2*)	TNIK,PASK,TPT1,FBLN7,ITGB2-AS1,FLT3LG,TCF7,MAL,BCL11B,CD4,CD5,TBC1D4,CD6,ICOS,PIM2,LAT,TIAM1,LINC00892,EML4,PHACTR2-AS1,CAMK4,CD28,TNFSF8,FAM102A,DUSP16,LEPROTL1,CD40LG,SUSD3,DGKA,CRYBG1,PBX4,SPOCK2,CCR4,CCR8,SARAF,TNFAIP3,PIK3IP1,FYB1,AQP3,F5,SLAMF1,PBXIP1,TESPA1,IL6R,SEPTIN6,ITK,IL7R,FAAH2,MAF,FOXP3
**CD8_Tcell_AvivRegev**	(*2*)	CD84,IFNG,PCED1B,ADORA2A,DNAJB1,PDCD1,BICDL1,PAG1,NELL2,PTPN7,CD3E,TNFRSF9,TTC24,CD8A,THEMIS,KLHL28,CD8B,TIMD4,TIGIT,TOX,MCOLN2,RNF19A,CD27,MAP4K1,ZBED2,MIR155HG,TNIP3,GZMK,CRTAM,LAG3,SIRPG,CLEC2D,CXCL13,ASXL2,JAKMIP1,GPR171,ASB2,ITGA4,CCDC141,ITGAE,CBLB,SIT1,TTN,SNAP47,LYST,TMEM155,ITM2A,RAB27A,MIAT,PRDM1
**CD8_Tcell**	internal_derived	KLRK1,CD7,CD8A,CD8B,PRF1,GZMH,GZMA,GZMB,XCL2,LAG3,ZNF683,CCL5,GNLY,NKG7,FASLG,XCL1,KLRC1,TRDC,KLRD1,CTSW
**Cellcycle_G1S_AvivRegev**	(*2*)	RAD51,BLM,TYMS,CASP8AP2,RAD51AP1,CHAF1B,BRIP1,CDCA7,POLA1,CCNE2,TIPIN,PRIM1,DTL,FEN1,CENPU,EXO1,NASP,UHRF1,GINS2,MCM2,MCM4,UNG,MCM5,SLBP,MCM6,MSH2,HELLS,POLD3,CDC6,CLSPN,WDR76,RFC2,RRM2,RRM1,ATAD2,DSCC1,RPA2,USP1,UBR7,GMNN,E2F8,PCNA,CDC45
**Cellcycle_G2M_AvivRegev**	(*2*)	CDCA2,NUSAP1,CDCA3,CKS1B,CKS2,NEK2,TUBB4B,RANGAP1,KIF23,CENPA,NDC80,CENPE,GAS2L3,CTCF,CENPF,GTSE1,UBE2C,DLGAP5,HJURP,TPX2,NCAPD2,MKI67,CKAP5,SMC4,CKAP2L,TMPO,HMGB2,PIMREG,G2E3,JPT1,NUF2,CDK1,HMMR,LBR,CKAP2,TACC3,CDC20,CDC25C,ECT2,CDCA8,TTK,TOP2A,PSRC1,KIF20B,KIF11,KIF2C
**Chemokine_Harlin**	internal_derived	CXCL10,CCL2,CCL3,CCL4,CCL5,CXCL9
**Cytolytic_MichaelRooney**	(*3*)	GZMA,PRF1
**DCSIGN_macrophage**	internal_derived	MRC1,FOLR2,PLTP,F13A1,CD209,CCL13,CLEC4G
**Dendritic_cells_Spranger**	(*4*)	ITGAE,IRF8,CXCL1,CCL3,CCL4,CCR5,BATF3
**Down_Treg_vs_Up_Teff_0h**	REACTOME	ST6GALNAC1,PCSK5,KRT72,ZNF609,NOG,DHRS3,LRRC24,CYSLTR1,SLC22A23,BACH2,PECAM1,OBSCN,NBL1,ARMH1,SOX8,CHI3L2,BCL7A,ZNF467,SLC15A3,ALS2CL,SLC22A17,PTK2,RNF157,ADD2,ME3,AXIN2,HDGFL3,ABCB1,KRT73,KLHL29,AKR1E2,DACT1,NELL2,NEO1,IFITM5,FCGBP,BPGM,SATB1,EDAR,GADD45G,MTUS1,KLF5,AK5,OSBPL5,ADGRE4P,AIF1,KCNQ1,DLG4,CACNA1I,CCL5,NKG7,DENND5A,FHIT,NME4,ADGRA3,C16orf54,PDZD4,CA6,NPAS2,VIPR1,ACSL6,COL6A1,TRABD2A,B4GALNT4,TCF7,SDK2,THEMIS,DSC1,ANK3,NRCAM,PGGHG,PLXDC1,BEND5,MMP28,MAN1C1,PPFIBP2,TMIGD2,WNT7A,WNT10B,ENC1,APBA2,HIPK2,DPYSL4,CHD7,CACHD1,CERS6,DLL1,CR2,BHLHE40,ANKRD55,SLC40A1,GPR160,GIPC3,AREG,SCML4,NLRP6,NOSIP,ADAMTS10,RHOB,RMRP,COL18A1,PLLP,NSG1,ZNF516,H1-0,SORBS3,CELA1,PRKAR1B,PROSER2,ADGRE1,SNTB1,SEC14L2,EPHA1,CYB561
**effector_vs_exhasusted_CD8_down**	(*5*)	CHL1,CFH,KCTD12,CFHR2,GABRR2,CRISP2,CYP2A6,TNFRSF9,CELF4,ERCC5,SPRED2,GCM2,CD244,AARD,HLA-DMA,MAP2,AUH,MYO6,SPP1,EFS,BET1,ACADVL,ENPP2,ADAM7,TRPC1,COPRS,PBDC1,PHLDA3,GDAP1,GATA2,MCAM,NAP1L2,ADGRG1,EXOSC8,SCRG1,SPOCK2,IRF6,SCAND1,IRS1,TLR7,SSBP2,RXYLT1,SLC30A1,PTGER2,WLS,F2RL1,SLC7A11,LIN9,GDNF,GTF3C4,LCLAT1,GCSAM,PTK6,RPRD1B,TNFRSF4,SMIM4,NSDHL,EOMES,ACSL1,EVI5,IFIH1,CKMT2,NEFH,DFFA,PTPN12,KANSL2,NEUROD1,CADM1,HOXC6,CLCA1,COCH,MDN1,PER2,PTPRJ,PIK3C2G,SUB1,YAP1,AFP,NFIB,SCN1A,RIN2,NRK,SCN7A,VAMP7,MITF,NQO1,AHR,SYT1,KCNAB1,FGF6,POLR1B,PNRC1,PLA2G10,NKX2-2,TWSG1,GNAO1,TUG1,STRA6,CPSF2,FHL1,INCA1,GSTO1,TAPBP,IMMT,METAP2,MTRF1L,VCAM1,NOTCH4,AGAP1,NELFE,DPP7,TBX15,CYP4V2,CARM1,GPLD1,GPM6B,H3C7,TCF4,ZNF821,RBM15,DDIT4,TMEM150A,MAGEL2,REXO5,COL19A1,SPOUT1,HTRA2,WFS1,SCAMP1,ANXA3,PCLO,CANX,POLR2C,DUSP6,NR4A2,CXCL13,ZFP28,PRXL2A,CCRL2,CXCL14,POU2F1,CPA3,ATP5MF,TM2D3,APP,SIX1,NKIRAS1,TERF1,C16orf72,RGS10,RGS16,RHAG,GSTM3,SLC6A4,LHCGR,HINFP,MSX1,NDUFA13,GMCL1,FRK,TGM2,CLDN11,ABCG1,FAM207A,OVGP1,IGF1R,CSF1,HMGA2,EFNB3,SLC12A2,ZNF35,P2RX4,MAP1S,RFLNB,CD22,EGR2,MRPS2,AOPEP,KIAA1217,NCAN,MRPL48,H1-4,DOCK7,CELA1,ZNRF1,PLEKHA1,PAWR,PAX1,IL1A,TM2D1,TM4SF1,ATP2A2,EPCAM,EIF2AK2,HAO2,ZRANB1,H19,SMAD1,PHLDB2,ZNF239
**effector_vs_exhasusted_CD8_up**	(*5*)	PRPSAP1,PALD1,CDKN2D,ATP6V0C,PTTG1,ABCA2,ATP6V0B,LRWD1,DYM,ATP5PO,REPS1,CCNDBP1,LGALS9B,SWAP70,DCTN5,RPN1,SETD6,EIF4A3,GDAP2,HIPK1,ACP5,BCKDK,PSMB2,PSMB3,MBP,SELENOH,TMC6,ETS1,STK38,DGKA,MTMR1,FAM89B,SRP68,DAP,SRPK1,GOLM1,TWF2,SMIM20,DBI,PSMD13,LYSMD1,PLD3,CMAS,ITGAL,ITGAX,IL18RAP,DHRS1,NDUFB6,RSU1,EIF6,HMCES,LDAH,ST13,HNRNPAB,ITGB7,DPM2,TUBA3C,PLAC8,SEPTIN6,DUS1L,KIAA2013,AK3,AXIN1,LSM1,B4GALT1,BRAP,EFTUD2,TBCB,BLVRA,CCR2,PTPN6,MFNG,ZIK1,FAM117A,BNIP3L,TXNL4A,TSPAN31,SATB1,UBE2H,FCGR2B,RNPS1,PIK3CD,KCNJ8,EIF2B4,EIF3L,EIF2B5,TMEM147,SCP2,AP1M1,LSM4,ANAPC16,CAB39L,IL17RA,AURKAIP1,FGFR3,TMEM223,EBNA1BP2,HSD11B1,SEMA4A,DNM1,RMC1,FHL2,DDX41,TIMM44,ANAPC5,MTCH1,KLRC1,KLRD1,C8orf33,VAV1,RABGGTA,YIPF3,UBE2Z,NSMCE1,RAD17,GPC1,DPEP1,DNAJB1,SLC66A2,ISYNA1,CIB1,DEAF1,RPS6KA4,UBQLN1,WARS1,USP22,DCPS,PIM2,MLX,GIT1,TMEM45A,HIKESHI,CALU,LAMTOR5,ANXA1,ANXA6,ICAM2,MKNK2,JKAMP,MIEN1,MACROH2A1,CORO1B,XPNPEP1,IFITM10,CPT2,CTSA,PPIB,PCGF5,SLC1A5,ENTPD4,CSRP3,WTAP,PPIF,PNPO,KLRK1,CDC37,RNF14,GSTT2,ORC5,ARL4C,USP5,RNF167,PDIA6,ARFRP1,TIAM1,LAMTOR4,FEZ2,GCAT,TMEM208,EIF2S1,PKP3,SSNA1,COMMD7,CHFR,GRAMD2B,KLK8,PRKAG1,GLIPR2,ZFYVE19,RACGAP1,ELAVL1,RORA,PRKCH,SMPD1,BSCL2,MRPL34,EIF3B,HADHB,RPP25L,LY6H,CTSD,CMTM7,LEF1,KLRG1,HERPUD1,SNX1,HCLS1,PHTF1,MTSS2

**LRRC15_fibroblast**	(*6*)	MMP11,CTHRC1,AEBP1,COL5A2,ITGA11,THBS2,C1QTNF3,COL10A1,COL11A1,COL12A1,LRRC15
**GOLDRATH_EFF_VS_MEMORY_CD8_TCELL_DN**	(*7*)	DUSP12,ACP6,MRPL23,TRAF3IP2,USP18,TRAF1,METTL3,ABCA2,TRAF5,ZBTB20,RPL39,MAN2C1,ZNF274,RPLP0,RPLP1,ACADM,NCKAP1,RRP1B,PDGFB,SUSD6,PDK1,SHISA5,DBNDD2,ACP5,RPS7,RPS8,MBP,TMC6,TTC14,ASH1L,DGKA,GBE1,RPS16,LYSMD2,SNRK,RPS18,PTPN22,RPS19,TNIP1,BCKDHB,RPS23,BCL2,LYSMD1,PSME2,GMIP,DBP,NDUFA7,C12orf57,EZH1,RTKN,TTC3,SIT1,MECP2,SESN1,ADCY7,DDIT3,STAT4,WDR43,SPICE1,ITPR2,NEDD4,KLF2,EOMES,TXK,RASSF5,JUN,EPS15L1,MFNG,PHLDB1,VKORC1,EML5,WDR75,PDRG1,CANT1,SUPT6H,SURF2,KCNJ8,PURA,CRLF3,COQ9,RPL19,GNL2,DIAPH2,TSEN34,POLR1B,PLEKHA5,KLF17,ITM2C,PISD,CCR7,SIPA1L2,HSF1,QDPR,WDR13,FAM8A1,HSD17B8,NOTCH4,PAQR7,SH3BP5,MCOLN2,CYP4V2,SELL,TLK2,CA12,RAD52,CCNL2,YPEL3,ABHD14A,EVL,SEMA4B,TCF7,ZFP90,ZEB1,ADGRG3,SLC11A2,NEDD4L,BCL2L11,CHCHD7,EMB,MAP4K3,SLC26A2,PRSS12,IAPP,TAF1C,TAF1A,ANKRD10,RTTN,TRMT1,TTC27,RABAC1,B3GALT6,ABCC5,LAMP1,SIAH1,SETD4,CST7,ICE1,BRD3,RPL14,DDX51,XPC,IFITM10,RPP14,XRCC5,ELP3,TESK1,ZNF692,PPP1R14B,C6orf136,UBLCP1,DALRD3,ADAM22,RGS10,TMED4,MTFR1L,POLR3A,MLYCD,PPOX,NECAP1,CCND2,SIX6,ARL4C,RNASE4,GTF2I,DPH5,CD7,AKAP9,NSG2,GSTK1,RFLNB,NACC2,TSR1,TK2,GALNT11,RASA4,WDR92,SBDS,ZKSCAN3,ZFP1,PRKACB,SLTM,C5orf51,PLEKHA1,COX7A2L,CIPC,LTB,EIF2AK4,PRMT3,H2AJ,FAM189B,RERE,HSD17B11,SLC12A7,IL4R,CD96,IL6R,CTSW,IL6ST,IL7R,TDRP
**GOLDRATH_EFF_VS_MEMORY_CD8_TCELL_UP**	(*7*)	NUSAP1,CDCA3,CDKN2C,CDKN3,ATP6V1A,PDCD1,IFI27L2,MYADM,SNX3,MIS18BP1,GALNT3,CD244,CENPA,INCENP,CISD1,PCLAF,PDK3,VPS45,PSMA5,SQLE,DLGAP5,PERP,CKAP5,KIF4A,HMGB3,IRF4,NUP62,ISG20,TKTL1,CHEK1,DBI,MCM10,LXN,NR4A1,RRM1,RRM2,EZH2,H3-4,DCK,ADAM8,ITGAX,TTK,DHRS1,GEM,CMC2,PLAC8,AK3,FGL2,SPDL1,NRP1,CDC45,SYCE2,TXN,TYMS,S100A4,GGH,DOCK5,AURKA,S100A8,TUBA1B,TYROBP,S100A10,CKS1B,CKS2,TUBB3,CCR2,NEK2,SERPINB9,NDRG1,GABARAPL1,CLCN5,BRCA1,TUBA1A,UBE2N,HP,IFITM3,ANLN,CD99,FDFT1,UCHL5,TIPIN,DHFR,BUB1,SNX10,MKI67,NFYB,ASPM,CBX1,SYPL1,UCK2,C3,TMEM97,BATF3,TAGLN2,DNAJC1,TACC3,DNA2,IQGAP3,FKBP5,TMEM50B,KIF11,CA2,KIF22,KIF2C,KPNA2,RAD51,RNASEH2B,RAN,KIF23,ATP5IF1,TCF19,WEE1,F2RL3,LITAF,RBL1,POLA1,ANXA1,ANXA2,CIP2A,POLE2,CHAF1A,DUSP2,FPR2,CAPG,PRDX4,TUBA1C,DTL,SUN1,ANXA4,LAG3,TMEM14C,IRF8,MPEG1,CASP3,XBP1,SIVA1,BIRC5,YBX3,STMN1,TSPAN32,IDI1,PRC1,SMC2,RFC5,FANCM,NCAPH,ECT2,CDCA8,BHLHE40,GMNN,DEGS1,LGALS1,E2F8,LGALS3,RHD,CRMP1,CCNA2,STT3B,KIF20A,IFNG,CCNF,LIG1,RAD51AP1,MT1E,GTF2B,MRPL18,MT2A,NUDT4,LMNB1,UBE2T,EGR1,TK1,ASF1B,CCNB2,PRIM1,MTM1,GZMA,GZMB,GZMK,H1-0,H2AX,H2AZ1,SMARCC1,TMPO,LSP1,ROM1,CDK1,SUZ12,EMP1,MAPK6,CDC6,CDC20,EMP3,LYN,CDC25C,KLRG1,PLK4,TOP2A,MAD2L1,AURKB
**HARRIS_HYPOXIA**	(*8*)	CDKN1A,CDKN1B,HDAC9,EPO,HGF,HIF1A,HK1,HK2,PDGFB,GAPDH,SPP1,HMOX1,PFKL,PFKP,F3,PGF,PGK1,PTGS2,DDIT3,AK3,LRP8,STC1,BIK,TXN,ADM,JUN,BNIP3,BNIP3L,SAT1,CD99,NFKB1,MIF,PKM,FGF3,CCL2,PLAUR,TAGLN,MMP13,ALDOA,VEGFA,CA9,CA12,VIM,COL5A1,FLT1,ANGPT2,FOS,L1CAM,APEX1,CP,XRCC5,TEK,LDHA,BHLHE40,TF,SLC2A1,EDN1,EDN2,SLC2A3,TFF3,TFRC,TGFA,TGFB1,TGFB3,CCNG2,TGM2,TH,IGF2,IGFBP1,IGFBP2,IGFBP3,P4HA1,FTL,RP1,ENO1,ENPEP,IL6,EPAS1,XRCC6,CXCL8,PRPS1
**HIF2A_Sensitivity_predictive_signature_Brugarolas**	(*9*)	C1QL1,SLC6A3,PKNOX2,POSTN,EPO,TPST2,TMEM30B,NPTX1,IGFBP1,CHRDL2,INHBB,AVPR2,CXCR4,WFIKKN1,GLI1,LOX,FAM180A,KNDC1,NFASC,SORCS3,HRH2,CPE,RDH13,CHST1,PTHLH,EPAS1,GFRA2,HSPB7
**HIF2A_target_genes**	internal_derived	EPO,EGLN3,CCND1,SERPINE1,ANGPTL4,NDRG1,IGFBP3,TGFA
**IFNG_10gene**	(*10*)	IFNG,IDO1,CXCL11,CXCL10,PRF1,CCR5,HLA-DRA,STAT1,GZMA,CXCL9
**IFNG_28gene**	(*10*)	IFNG,PDCD1,CD2,CD3D,CD3E,SLAMF6,PTPRC,IDO1,CIITA,CXCR6,TAGAP,CXCL10,PRF1,HLA-DRA,GZMA,GZMB,GZMK,CXCL9,HLA-E,LAG3,CXCL13,CCL5,CCR5,NKG7,ITGAL,CXCL11,IL2RG,STAT1
**IL18_PBMC_24HR_UP**	internal_derived	IFNG,SLC41A2,DOK5,TUBB6,NEMP1,P4HA2,SYNPO2,IL15RA,LIMK2,TMTC1,TNFRSF9,CSF2,FN1,P2RY12,FEZ1,INHBA,CD80,RHEBL1,CASP5,GPR84,CD274,C1QB,CCL1,CCL3,ELAVL4,MYO1B,CCL7,ALAS1,CCND1,HSD11B1,CCL19,CHI3L1,SUCNR1,IL1A,FAM20A,NEXN,FMNL2,F3,RGS1,RGS16,CCND2
**IL1B_extended**	internal_derived	IL1B,IL1R1,IL1RN,IL1R2,IL6,IL18BP,IL18,CXCL8
**IL1b_macrophage**	internal_derived	IL1B,CXCL1,CXCL2,CXCL3,IL6,NLRP3
**IL1B_PBMC_24HR_UP**	internal_derived	IL10,IL13,NRG1,SYNPO2,ABCA1,IL17A,HTRA1,EREG,MSC-AS1,KREMEN1,MAOA,PDGFRA,SPP1,MYO10,SYT17,CLEC5A,ADAM9,VNN3,CYP27B1,TNIP3,LOC440896,DNER,GPR84,ETV5,SLCO4A1,BCAT1,ITGA1,PPP1R17,CHI3L1,EPB41L3,ADGRE2,MSANTD3-TMEFF1,SLC30A4,IL17F,HPSE,SLC7A11,MS4A7,PMEPA1,CRLF2,PTGS2,TUBB2A,ITGB8,CD163,LINC01093,SLC39A8,STC1,EHD2,PRR16,SLC41A2,S100A9,QSOX1,MET,S100A12,NEFH,S100B,MS4A4A,GJB2,KL,GSDME,NF1,TREML4,FCAR,MUCL1,IL1R2,VNN1,SPHK1,SMPDL3A,ARNT2,GLUL,TMEM150C,CCL2,C3,C3AR1,MAFB,PLAUR,GNA15,CCL8,ATP13A3,CCL7,FLRT2,KIFC3,MMP1,CCL19,CCL20,MMP7,MMP9,MERTK,CCL23,STEAP1B,MMP14,CXCL6,CXCL5,MMP19,PLOD2,HS3ST1,LILRA5,PID1,SDC2,RAB13,PLTP,NOTCH3,LILRB4,ZMYND15,CA12,FNDC3B,P4HA2,LILRA1,SEMA3C,LILRA3,LILRA2,DMXL2,SLC4A8,ANG,SLAMF9,FN1,ANPEP,PHLDA1,PPP1R3B,OLFML2B,PROCR,SPSB1,HBEGF,DUSP1,WNT5A,FPR2,DUSP6,SOCS3,LILRA6,CXCL13,CASP5,CP,PAPLN,MYO1B,CPD,EFCAB3,PNKD,SKIL,NRIP3,CXCL1,CXCL2,CXCL3,SLC1A2,RGS1,VMO1,AQP9,ABCC6,LEPR,AREG,TFPI,CEMIP,PDPN,THBD,THBS1,FSD1L,RNASE2,SH3PXD2B,NAMPT,RNASE4,EFNA5,SLC11A1,LRRC25,LOC401312,CD14,LACC1,CD80,VCAN,SLC22A4,IL22,FTH1,TREM1,FCRLB,STEAP3,ZNRF1,FAM124A,RAI14,LTBP2,LIMCH1,HAMP,EMP1,ADGRE1,IL1A,IL1B,CTTN,FAM20A,OLIG2,CTSL,TRIB1,IL6,TCEAL9,CXCL8,SLC16A10
**Immune_Bcell_KM**	(*11*)	BLK,SPIB,TNFRSF13C,CXCR5,CD19,MS4A1,TNFRSF13B,CD79B

**Immune_BcellMemory_KM**	(*11*)	TNFRSF17,MZB1,JCHAIN,IGHD,IGHG1,IGLL5,FCRL5
**Immune_Chemokine_myeloid_KM**	(*11*)	CCL2,CCL3,CCL4,OSM,CCL8,CCL4L1
**Immune_Chemokine_neutrophil_KM**	(*11*)	CXCL5,CXCL1,CXCL2,CXCL3,CXCL8,CCL20
**Immune_Cytotoxic_KM**	(*11*)	FASLG,PRF1,CCL5,NKG7,GZMH,GZMA
**Immune_DC_Langerhans_KM**	(*11*)	CD207,CD1A,CD1B,CD1C,HLA-DQB2,CD1E,CCL17,FCER1A,CLEC10A,CCL22
**Immune_DC_like_KM**	(*11*)	LST1,PTCRA,LSP1,DOK2,LGALS9
**Immune_Fibroblast_KM**	(*11*)	GXYLT2,COL3A1,ADAMTS12,POSTN,CCN4,COL5A2,COLEC12,BNC2,COL6A3,MFAP5,FAP,FNDC1,ITGA11,COL8A1,THBS2,COL10A1,VGLL3,FBLN2,KCNE4,SFRP2,MSC-AS1,LGI2,ASPN,SULF1,TNN,CTHRC1,MEDAG,C3orf80,CPXM1,CLMP,MMP2,GREM1,TNFAIP6,HAS2,GPC6,EMILIN1,RGS4,DKK2,EDNRA,ALPK2,LRRC15,COL1A1,COL1A2
**Immune_IFNG_KM**	(*11*)	IFNG,CXCL11,GBP1,CXCL10,CXCL9
**Immune_M2_macrophage_KM**	(*11*)	IL10,MRC1,MS4A4A,FOLR2,LILRB5,F13A1,CD209,CD163L1,CD163
**Immune_Macrophage_KM**	(*11*)	CD84,MSR1,CYBB,HAVCR2,LST1,SIGLEC7,CSF1R,FCER1G,FCGR2A,FCGR3A,GPR34,CD86,LILRB2,FPR1,VSIG4,CD68,C1QA,C1QC,C1QB,TLR7,SIGLEC1,ITGAM,ITGAX,LILRB1,TLR8,LILRB4
**Immune_Mast_cell_KM**	(*11*)	CDK15,TPSD1,RHEX,TPSAB1,HPGDS,SIGLEC17P,CPA3,SIGLEC6,RGS13,TPSB2,HDC,MS4A2
**Immune_Osteoclast_like_KM**	(*11*)	HS3ST2,DNASE2B,ATP6V0D2,CCL18,DCSTAMP,CHIT1
**Immune_Phagocytic_CLEC9A_KM**	(*11*)	GAPT,SIGLEC8,CX3CR1,PLD4,CLEC9A
**Immune_PMN1_KM**	(*11*)	PROK2,FCGR3B,FFAR2,CXCR1,CXCR2
**Immune_PMN2_KM**	(*11*)	LILRA5,CD300E,MEFV,FPR1,FPR2,FCAR
**Immune_PMN3_KM**	(*11*)	TREM1,IL1RN,AQP9,PLAUR,CCL7,SLC11A1
**Immune_signature_ESTIMATE_Yoshihara**	(*12*)	CCDC69,CYBB,IL10RA,GIMAP4,CD52,IL32,HLA-B,ARHGAP15,HLA-DMA,HLA-DPA1,RASSF2,HLA-DPB1,LILRB2,HLA-DRA,CD300A,HLA-E,HLA-F,HLA-G,TCIRG1,GBP1,GBP2,EVI2B,NCF2,NCF4,IKZF1,BCL2A1,GMIP,ITGA4,LY96,ITGAL,PTGER2,ADAM8,PTGER4,IL18RAP,ITGB2,LILRB1,ADCY7,ITK,FGL2,LAPTM5,S100A8,S100A9,TYROBP,PTPRC,PTPRCAP,FCER1G,PTPRE,CLEC4A,VNN2,TPP1,RABGAP1L,NFKBIA,GLRX,LPXN,PVRIG,IFI30,P2RY14,MAFB,CCL5,CD302,NKG7,BIN2,CCR7,FGR,PLEK,GIMAP6,TAP1,MFSD1,KLRB1,MNDA,RAB27A,VAV1,ARHGEF6,ALOX5AP,CLEC2B,RAC2,DOCK2,SELL,LST1,SELPLG,FLI1,TPST2,MICAL1,GPR65,WIPF1,SH2B3,AOAH,LAIR1,GMFG,CASP1,IRF8,GNLY,CST7,SAMHD1,PSTPIP1,LCK,LCP2,SLA,RGS1,ZAP70,LGALS9,MSN,TGFB1,KLRK1,RHOG,ARHGDIB,CORO1A,RHOH,CD2,CD3D,CD247,RNASE6,CD27,PRF1,SRGN,CD37,GZMH,GZMB,GZMK,MYO1F,CD48,CD53,CSTA,CD69,CD74,LSP1,LTB,TRAF3IP3,TNFAIP3,TNFRSF1B,EMP3,LYZ,FYB1,IL2RB,IL2RG,IL4R,HCK,CTSS,HCLS1,IL7R,NCKAP1L
**Immune_Tcell_KM**	(*11*)	SH2D1A,TRAT1,CXCR6,CD2,CD3D,CD3E,CD3G,CD247,GZMK,PYHIN1,CD8A,CD8B,SIRPG
**Immune_Treg_KM**	(*11*)	LAIR2,TIGIT,CXCL13,ICOS,LTA,ZBED2,CTLA4,TNIP3,CRTAM,FOXP3
**Immune_TypeI_IFN_KM**	(*11*)	RSAD2,ISG15,IFIH1,IFIT2,IFIT1,OAS2,BST2,USP18,IFI6,SP100,IFI44L,MX1,MX2,OASL
**IPRES_DEG_down_WillyHugo**	(*13*)	OLIG1,TRAF3IP2,CDH1
**IPRES_DEG_up_WillyHugo**	(*13*)	PKDCC,ALDH1L2,RASL11B,MFAP2,MEX3B,TDRD10,CILP2,SLC45A1,FOXC2
**ISG_IFN1_HuayangLiu**	(*14*)	TDRD7,IFIH1,PNPT1,USP18,SAMD9L,NMI,RSAD2,TRIM25,TRAFD1,DHX58,UBE2L6,EPSTI1,GBP4,ISG15,CXCL10,BST2,OGFR,DDX60,OASL,CASP1,CMPK2,TRIM21,IRF7,ISG20,PSME2,HERC6,PARP14,TRIM14,SP110,IFI35,IFIT2,EIF2AK2,ADAR,RTP4,IFIT3,STAT2,MX1,PARP12
**Lineage_basophile_AvivRegev**	(*2*)	CD44,ANPEP,CD63,CD69,IL3RA,LAMP1,CCR3,ENPP3,ICAM1,TLR4

**Lineage_Bcell_AvivRegev**	(*2*)	BLK,CD93,PDCD1,CD2,TNFRSF9,CD5,FCER2,CD19,MS4A1,CD22,CD80,CD86,CD40,TNFRSF13C,TNFRSF13B,CD69,CD70,CD79A,CD79B,PAX5,SDC1,TNFSF4
**Lineage_CAF_AvivRegev**	(*2*)	DCN,COL6A1,COL6A2,COL6A3,FAP,COL1A2,COL1A1,THY1
**Lineage_CD4T**	internal_derived	TRAC,CD3E,CD3D,CD3G,CD4
**Lineage_CytotoxicT_AvivRegev**	(*2*)	IFNG,CCL3,CCL4,PRF1,CST7,NKG7,GZMA,GZMB
**Lineage_endothelial_AvivRegev**	(*2*)	SELE,MCAM,TEK,ENG,ITGB3,CDH5,KDR,PROCR,CD34,FLT4,VCAM1,PECAM1,VWF,ICAM1
**Lineage_eosinophil_AvivRegev**	(*2*)	CD244,FCER2,CD53,S100A9,SIGLEC10,SIGLEC8,CCR1,C3AR1,CCR3,CXCR3,CD52,PTGDR2,C5AR1,ITGA4,IL9R,FUT4,LAIR1
**Lineage_exhaustedT_AvivRegev**	(*2*)	TIGIT,HAVCR2,PDCD1,CTLA4,LAG3
**Lineage_immune**	internal_derived	CD14,FCGR3A,FCGR3B,CD68,CD79A,CD1C,CLEC4C,TRAC,CD3E,KLRF1,CD4,LILRA4,CD8A,CD8B,FOXP3
**Lineage_macrophage_AvivRegev**	(*2*)	FCGR1A,CD14,ITGAL,ITGAM,ENG,ITGAX,CD68,CD80,CD86,LILRB4,LAMP2,CD33,CCR5,CD163,TLR2,TLR4,CSF1R,FUT4
**Lineage_mast_cell_AvivRegev**	(*2*)	ENPP3,KIT
**Lineage_MDSC_AvivRegev**	(*2*)	CD1A,CD1B,CD1C,CD4,HLA-DOA,HLA-DOB,CD80,CD86,HLA-DRA,HLA-DRB1,HLA-DRB5,HLA-DRB6,CD207,CD40,CCR7,CMKLR1,ITGA4,CD83,LY75,ITGAM,ITGAX,CD209,PDCD1LG2,NRP1
**Lineage_Megakarocyte_AvivRegev**	(*2*)	CD9,SELP,ITGAV,ITGB3,PECAM1,ITGA2B,GP1BA
**Lineage_Myeloid_DC_AvivRegev**	(*2*)	CD1A,CD1B,CD1C,CD4,CD80,CD86,CD207,CD40,CCR7,CMKLR1,ITGA4,CD83,LY75,ITGAM,ITGAX,DCX,CD209,PDCD1LG2,NRP1
**Lineage_naiveT_AvivRegev**	(*2*)	LEF1,SELL,CCR7,TCF7
**Lineage_Neutrophil_AvivRegev**	(*2*)	CEACAM8,FCGR1A,SELL,ANPEP,ITGAM,CSF3R,CD14,ITGAX,CD33,CXCR2,PECAM1,C5AR1,CXCR1,MME,TLR2,FUT4
**Lineage_neutrophil_Danaher**	(*2*)	CSF3R,FCGR3B,SIGLEC5,S100A12,FCAR,FPR1,CEACAM3
**Lineage_NK_AvivRegev**	(*2*)	CD244,KLRK1,ITGAM,ITGAX,IL2RB,CD69,KLRB1,NCAM1,KLRC1,B3GAT1,KLRD1,SLAMF6,SIGLEC7,ITGA2,KLRA1P,NCR1,SLAMF7
**Lineage_pDC**	internal_derived	CLEC4C,LILRA4,IL3RA
**Lineage_stromal_AvivRegev**	(*2*)	THY1,PDGFRA,TIMP1,TIMP2,PDGFRB,PECAM1,ICAM2,TLR2,TLR1,ICAM3,TLR3,TLR4,ICAM1,MME,MMP1,MMP2,ITGA4,MMP9,KIT,ITGB1,MADCAM1,VCAM1
**Lineage_Tfh_AvivRegev**	(*2*)	CD84,CXCR5,SLAMF1,PDCD1,TNFSF4,IL6R,CD3D,CD3E,CD3G,STAT3,CD4,ICOS,BCL6,CD40LG
**Lineage_Th1_AvivRegev**	(*2*)	IFNG,IFNGR1,HAVCR2,TNFSF11,IL2,IL12A,IL12B,DPP4,CCR1,IFNA1,KLRD1,CXCR3,CCR5,TNF,CD4,TBX21,CSF2
**Lineage_Th17_AvivRegev**	(*2*)	IL1R1,IL17F,KLRB1,CCR4,CD3D,CD3E,CD3G,LINC-ROR,STAT3,IL22,CD38,IL17A,IL21,CCR6,CD4
**Lineage_Th2_AvivRegev**	(*2*)	GATA3,IL1R1,IL10,HAVCR1,IL13,IL4,CXCR4,IL5,CCR3,IL6,CCR4,PTGDR2,CCR7,CCR8,CD4,ICOS,CSF2
**Lineage_Th22_AvivRegev**	(*2*)	AHR,CCR10,CCR4,CCR6,CD3E,CD3D,CD3G,CD4
**Lineage_Th9_AvivRegev**	(*2*)	GATA3,IRF4,CD3D,CD3E,CD3G,CD4,STAT6
**Lineage_Treg_AvivRegev**	(*2*)	IL2RA,FOXP3
**M1_macrophage_1**	(*1*)	IFNG,CXCL13,CXCL11,IL23A,CXCL10,CXCL9,CD86,CCL5,CCL8,STAT1,TNF,CCR7,CCL19
**M1_macrophage_2**	internal_derived	CXCL11,CXCL10,STAT1,CD38,CXCL9

**M2_macrophage**	(*1*)	TGFB1,MSR1,PPBP,IL1A,CD14,IL1RN,S100A8,MRC1,S100A9,S100A12,PF4,C5AR1,SAA1,CD163,CXCL8,CXCR1,CXCR2,ARG1
**MPAS_Wagle**	(*15*)	ETV4,ETV5,SPRY2,EPHA2,CCND1,DUSP4,SPRY4,DUSP6,EPHA4,PHLDA1
**NK_function**	internal_derived	TYK2,IFNG,IFNGR1,JAK1,PRF1,GZMH,GZMA,GZMB,GZMK,GZMM,KIR3DL3,GNLY,KIR2DL1,KIR2DL3,KIR2DS1,KIR3DL1,KIR3DL2,KIR3DS1,STAT1
**NK_lineage_Nanostring**	Based on Nanostring panel annotation	KLRK1,KIR3DL1,KIR3DL2,KIR3DL3,KIR3DS1,IL12A,IL12B,KLRG1,KLRB1,KLRC1,KLRC2,KLRD1,KLRF1,KIR2DL1,KIR2DL3,NCR1,KIR2DS1
**NLRP3_Kawana**	(*16*)	RPL21,GADD45B,SMG1,HCAR2,HIP1,SNHG12,RPLP2,TNFSF9,CARMIL3,AGPAT4,LOC645188,NR4A1,CD83,PPP1R15A,WNK1,PTGER4,F3,PTGS2,ZEB2,LOC728715,JUNB,TAS2R14,RND1,SAT1,CLCN6,NPIPB6,IER3,CLK1,KCNJ2,NFKBIA,DKC1,CCL3,TAS2R46,CCL20,TAS2R50,PLK3,TAF1D,DNHD1,ZC3H12A,TBC1D30,PTBP2,MAP3K8,FOS,FOSB,DUSP1,DUSP2,SOCS3,NR4A2,NFKBID,RAPGEF1,NFKBIZ,NR4A3,CHST11,CMSS1,NR1D1,CXCL2,IL23A,CCL3L3,ZFP36,LARP7,FRY,EGR1,EGR2,EGR3,C3orf35,EIF4A1,PDE4DIP,RASGEF1B,UBAC2,MACF1,PKN2,SLED1,HBG1,EPCAM,TRIM44,PCID2,CXCL8,MAP3K2
**Pan-F-TBRS_Genentech**	(*17*)	TPM1,COL4A1,TGFBI,ADAM19,IGFBP3,FSTL3,SEMA7A,RFLNB,SH3PXD2A,PXDC1,ACTA2,ACTG2,CCN2,TAGLN,CTPS1,ADAM12,TNS1,CNN1,HSPB1
**pDC_2gene**	internal_derived	CLEC4C,LILRA4
**ProgressivelyDown**	(*18*)	FAM153CP,ANKRD31,KCTD12,LDLRAP1,SEH1L,FAM86B1,KANSL1L,RPL30,LGMN,ZNF135,KRT72,RIPOR2,RAPGEF6,MAL,NOG,CYP2J2,HLA-DOA,JAGN1,PDK1,RPS3A,INPP5A,MRM1,GJB6,SERPINF1,NOL6,RBM26,NAP1L1,SCOC-AS1,GATM,C16orf74,ACTN1,KAT2A,DENND2D,TNFRSF10D,METAP1D,ZSWIM1,ACSS2,CHKA,BCL9,KDM6B,ARHGEF11,UBASH3A,NREP,FCHO1,SLC22A17,TESPA1,NDUFS2,GCSAM,ITPKB,KRT73,RPL10A,RPP25,JAK3,CHMP7,MEST,KLHL3,CEP68,PCYOX1L,PTPN6,SULT1B1,MYO15B,SLC8B1,FCGBP,ADPRM,STXBP1,CFAP92,UBE2E2,FCER1G,USP51,KCNQ5,EDAR,PTPRK,AEBP1,XPO6,ABRAXAS1,AK5,RAB43,CRLF3,EIF2B4,THG1L,ZNF506,KIAA1958,NSUN5P2,WDR89,KCNQ1,PYGB,AP1M1,CDNF,EEF2K,GNAI1,GID8,ZNF121,TAF4B,TACC3,NME1,TOP1MT,HS3ST3B1,HSF2,MMP19,ZBTB18,SMAP2,AKR1B1,TARBP1,TMEM220,CDCA7L,CNN3,FGD3,ADGRA3,RIC3,ANAPC13,MPI,USP53,PITPNM2,SFXN4,TCEA3,VIPR1,NSUN5P1,FLNB,RBM11,UXS1,TBC1D15,SDK2,SGTB,DSC1,PIM2,TIMD4,LRRN1,NRCAM,TAPT1-AS1,BEND5,BZW2,MAN1C1,PPFIBP2,KLF3-AS1,CARMIL1,NUCB2,NUMA1,NUP88,LARS1,APBA2,GRAP,ARHGEF18,GAL3ST4,NBEA,FAM117B,CACHD1,CERS6,ZNF101,OCRL,OCM2,NUDCD3,ALPK1,MLXIP,ZNF496,LDHB,TSEN2,TXLNGY,LINC01089,SFMBT2,AARSD1,GIPC3,NUP35,ZNF563,NMNAT3,AGBL3,ZFAND1,RHOH,MICU3,FAM153B,TTTY14,ACBD4,SUGCT,LMO7,RASA4,MED28,ZNF540,COA1,MRPS33,GPSM3,PRKAR1B,LRP6,SNORA54,TMPO,FOXP1,ATM,DEPDC7,UBASH3B,CCDC102B,LAS1L,PBX3,LEF1,ZNF542P,ETFRF1,DCBLD2,MINDY1,IL6ST,CLUHP3,RBM43,RASGRP2,SNORD109A,FCMR,SLC16A10
**ProgressivelyUp**	(*18*)	PIK3AP1,ADGRG5,ABHD17A,ABCA2,STOM,ABI3,MAN1A1,PRR5L,ABL1,CRIM1,TP53INP1,ERN1,MATK,TNIP3,ABHD6,PI4K2A,TCIRG1,C4orf50,TNIP1,ACTN4,KATNAL1,SCD5,ST6GALNAC6,ADAM8,F2R,FKBP11,MEF2D,ADCY9,RAB6C,MAP3K5,PYHIN1,FANCA,EOMES,PLEKHG1,KLRF1,ADRB2,AGO4,ATXN1,CD99,MAP1LC3B2,OSBPL7,AK1,CHST12,AFDN,SLA2,CCL5,LOC84214,WDFY1,ARAP2,ZC3H12A,EMB,ANXA2,ANXA2P2,FOSL2,ANXA5,PIK3R3,HCN3,PIWIL4,CHD9,GNLY,CST7,NAA50,FAS,FAM160B1,FASLG,SLC1A4,MXD4,ST6GALNAC2,S1PR5,CNPPD1,SLC6A6,SETD2,CXCR6,GNA13,GPR153,TBK1,FHOD1,MYO1F,GAB3,FUCA2,REEP4,SLAMF7,SLC4A4,STX11,PEA15,ATP2B4,MVD,MYBL1,SELENOT,NUGGC,SPIRE2,GGA2,GALK1,MBOAT1,MYL6,GALNT3,MYO5A,MYO6,NHSL2,TTC39C,TBC1D2B,MAP3K20,LPCAT4,WSB2,BCL2L1,PLXND1,GCNT1,ATG2A,IL18RAP,FRMD4B,GGT3P,IQGAP1,PRDM1,SHKBP1,BMPR1A,NEO1,NTNG2,NF1,PNPLA6,PELO,ARL6IP1,ST8SIA6,SYT11,NBEAL2,SYNE2,BTG3,JAZF1,TPRG1,C3AR1,APOBEC3C,DNAJC1,GNAO1,YWHAQ,HNRNPLL,TBCD,ANKS1A,NPC1,CACNB1,CACNB3,NRDC,C12orf75,EML4,SYNE1,KMT5A,CAPN2,ARHGEF12,RAPGEF1,GPRIN3,SH2D2A,ARHGAP35,RUNX3,CCDC167,TGFB1,TGFBI,TGFBR3,BROX,THBS1,CMIP,ZSCAN22,TARP,GYG1,CBFA2T2,TLN1,GZMA,TLR3,HEATR9,TMX4,CD58,CD68,CD69,PAM,CD151,DUSP10,TNFAIP3,TNFRSF1B,CLCF1,ATG4D,SP140,NRM,DLG5,HIP1,TRAF6,MSC,ACOT9,HLA-DQB2,CYTH3,HLA-DRA,CD300A,HLA-DRB5,DCBLD1,YPEL1,SLFN12L,B4GALT5,CADM1,KIF3B,GABARAPL1,SMIM14,CLIC1,PIK3CG,PIK3R1,EDARADD,AHNAK,PIP4K2A,PLEKHA5,CCR3,PLCD1,PLD1,MTCH2,LRRC75A,PMAIP1,APOBEC3D,RNF166,WIPF1,COPA,KLF6,RNF19A,WEE1,ICAM1,TBX21,CHST11,TMEM43,TSEN54,GTPBP1,IDS,IFI16,BABAM2,SOX13,TIPARP,PPP2R2B,FOXP4,PPP2R5C,PPP3CA,SPATS2L,LRP10,MICAL2,PRF1,SRGN,LPIN2,PPP1R16B,AUTS2,FAM53B,PRKCI,CTNNA1,SLFN11,MAPK1,ARHGAP18,IL2RB,IL12RB1,IL15RA,CLSTN3,PHACTR2,TBKBP1,GALM,TOX,SUSD6,TMEM94,TBC1D5,PIEZO1,SPCS2,TENT5C,IRF1,PTPN22,PTGDR,ULBP3,ZEB2,MXRA7,LRP8,APOBEC3F,ITPR3,TRANK1,C1orf21,ANTXR2,PTPRJ,PTPRM,SV2A,LPGAT1,DLG3,KRTAP5-2,GFPT2,KIR2DL3,DMPK,REEP5,PCBP4,DTHD1,KLRB1,KLRD1,RAB27A,RAB27B,CDCA4,PPP4R1,RALGDS,WIPI1,ST7,PARP8,DUSP2,DUSP4,LAG3,RASGEF1A,RDX,NR4A3,REL,GTDC1,LCP1,TYMP,RGS2,LGALS1,CALHM2,CLIP4,CNNM3,ARL4C,RASGRP1,LIMK1,LLGL2,FADS3,CDK2AP1,NETO2,SYTL3,PLXNC1,ZC3HAV1L,PLEKHA2,RORA,TMEM39A,KLRG1,MFSD10,JHY,UEVLD,MAF
**REACTOME_GLYCOLYSIS**	REACTOME	GPI,PPP2CA,PPP2CB,PPP2R1A,PPP2R1B,PPP2R5D,LOC642969,GAPDH,PKLR,PKM,PFKFB1,PFKFB2,PFKFB3,GAPDHS,PFKL,PFKFB4,PFKM,PFKP,ALDOA,ALDOB,ALDOC,PGAM1,PGAM2,ENO1,ENO2,ENO3,TPI1P1,PGK1,TPI1
**Stromal_signature_ESTIMATE_Yoshihara**	(*12*)	HGF,PDE2A,IGSF6,ATP8B4,RAMP3,ERG,PDGFRB,GIMAP5,ASPN,ENPP2,ZNF423,LRRC32,VSIG4,CLEC7A,ACTG2,TLR7,ISLR,SAMSN1,PCDH12,DCN,ITGAM,PTGER3,IL18R1,PTGIS,ZEB2,F13A1,NME8,HEPH,ITIH3,CD163,BGN,TNFSF4,CCN4,MS4A4A,ITGBL1,FAP,FBLN2,OLFML1,PLPPR4,TRAT1,FCGR2B,FCGR2A,SULF1,SPON2,SPON1,KCNJ8,RASGRP3,BTK,CXCL9,SERPING1,AIF1,C1QA,C1QB,DIO2,LMOD1,COL14A1,CCR1,C3AR1,KDR,MMP3,MS4A6A,LY86,ITM2A,CXCL12,VCAM1,FOXF1,APBB1IP,CD200,COL1A2,COL3A1,TFEC,COL6A3,COL8A2,COL10A1,FMO1,MXRA5,SFRP4,COL15A1,RARRES2,TCF21,COMP,CILP,CD248,PLXDC1,SGCD,OLFML2B,WNT2,ECM2,FPR1,DDR2,CH25H,COX7A1,CXCL14,NOX4,COL5A3,SP140,ADAMTS5,RUNX1T1,FASLG,EDIL3,ADAM12,ADGRA2,RGS4,EDNRA,ZFPM2,EGFL6,ITIH5,LDB2,

		MSR1,CD93,MFAP5,THBS2,RSAD2,IGF1,TENM4,CSF1R,SCUBE2,CD14,ABCA6,PLXNC1,CD86,CD33,TNN,FRZB,TLR2,AOC3,PAPPA,ARHGAP28,SIGLEC1,PRKG1,GREM1,LUM,IL1B,SH2D1A,CDH5,ENPEP,PIK3R5,EMCN,LRRC15,HDC,MAF
**suppressive_TME**	internal_derived	TGFB1,IL10,TGFB2,CCL17,ARG1
**suppressorPMN_OPM**	internal_derived	TGFB1,MPO,DEFA1,IL13RA1,PRG1,CSF2RA,CSF2RB,CSF3R,FCGR3B,FCGRT,IL1R2,VNN2,RASSF2,CTSC,FPR1,ICAM3,CEACAM8,CD68,GCA,C3AR1,NCF2,IFITM2,MME,BCL6,MMP8,MMP9,GNB2,FUT4,IL1R1,CTSB,CTSA,ITM2B,ITGAM,TALDO1,IL1RN,IL18RAP,MNDA,RGS2,AQP9,SOD2,CXCR2,ARG1
**TAM_ShirleyLiu**	(*1*)	CDR2L,PCED1B,CD1A,CD1E,ABCC4,RNASE1,RAB33A,RGS18,FOXQ1,RAMP1,FCER1A,DUOXA1,MAOA,ZNF366,RRS1,MAP4K1,SYT17,IPCEF1,RAB30,QPRT,ESPNL,GPT,PPP1R14A,GATM,FAM189A2,PON2,ITM2C,CCL13,SH3BP4,CCL17,DHRS2,CALCRL,CCL18,CCL23,PDGFC,MS4A6A,CCL26,GALNT18,PALLD,CRH,F13A1,IL17RB,RASAL1,ALOX15,FABP4,PARM1,MOCOS,CMTM8,STAB1
**IFNG_pathway (Tcell_inflamed)**	(*19*)	TIGIT,PSMB10,IDO1,CD274,CXCR6,CD27,HLA-DQA1,CD8A,CCL5,NKG7,HLA-DRB1,STAT1,HLA-E,CMKLR1,CXCL9,PDCD1LG2,CD276,LAG3
**TCMvsTEM_DOWN**	(*18*)	OSBPL5,CTNNA1,ST8SIA6,FASLG,AUTS2,GALNT10,THBS1,GNLY,B4GALT5,SH2D2A,MAN1A1,MYBL1,GALNT3
**TCMvsTEM_UP**	(*18*)	SFXN1,SELL,SESN3,CDH1,RGMB,TSHZ2,BMERB1,CEP68,HMGB3,TESPA1,IL6ST,SULT1B1,WNT7A,OCM2
**TGFb**	internal_derived	FOXS1,SOX4,PMEPA1,HEYL,FAP,ALOX5AP,COL1A1,TBC1D2B
**TLS_Fridman**	(*20*)	CXCL13,CXCL11,CXCL10,CCL3,CCL2,CXCL9,CCL4,CCL5,CCL8,CCL18,CCL19,CCL21
**TNvsTCM_DOWN**	(*18*)	NUGGC,CLSTN3,PHACTR2,TSPAN18,PRR5L,MBOAT1,GALM,CRIM1,MYO1C,ERN1,TOX,EPHA4,ACOT9,HLA-DRA,PIEZO1,DCBLD1,SLCO3A1,ETV6,HMGB3,IRF4,ACTN4,DGKH,CHEK1,CHN1,ADAM8,ITGB1,ZEB2,MAP3K1,MAP3K5,LRP8,PYHIN1,BMPR1A,AGO4,PTPRM,EDARADD,AHNAK,NBEAL2,SYNE2,ARID5B,GLUL,JOSD1,TPRG1,CCL5,CCR4,GNAO1,DTHD1,HNRNPLL,RAB27A,PMAIP1,ARAP2,KPNA2,MCOLN2,NPC1,EZR,SNORD115-26,NIBAN1,APOBEC3D,WEE1,RNF19A,SYNE1,FOSL2,TBX21,P2RY8,GPRIN3,CLDND1,CST7,USP46,SH2D2A,GPR183,ST8SIA1,FAS,FAM160B1,NCAPH,OGDH,SLAMF1,RGS2,SLC1A4,COTL1,ST6GALNAC2,TGFBR3,PPP2R2B,THBS1,TIGIT,LRIG1,TARP,NETO2,SYTL3,SNORD115-11,SNORD115-12,SNORD115-20,ENTPD1,GZMK,MYO1F,FTH1,AUTS2,CD58,PAM,FAM53B,IGSF9B,SLFN11,TNFRSF1B,ARHGAP18,CDH1,IL2RB,ATP2B4,STOM,MYBL1,IL9R,MAF
**TNvsTCM_UP**	(*18*)	KRT72,LGMN,TPST1,FLNB,SULT1B1,SDK2,MAL,IGF1R,NOG,UBE2E2,KCNQ5,TIMD4,EDAR,LRRN1,PTPRK,PPFIBP2,ENPP2,AK5,GJB6,NUCB2,APBA2,PAK1,GATM,GAL3ST4,NBEA,TNFRSF10D,METAP1D,MLXIP,DEPDC7,CHML,NREP,PRAG1,ZSCAN23,SLC40A1,CNN3,ADGRA3,AGBL3
**TNvsTEM_DOWN**	(*18*)	PIK3AP1,CLSTN3,ABCA2,PHACTR2,GGA2,MAN1A1,PRR5L,TBKBP1,ERN1,MYO5A,ACOT9,METRNL,MATK,CD300A,CYTH3,PIEZO1,TNIP3,MAP3K20,PI4K2A,TENT5C,APOBEC3G,PTPN22,ACTN4,PLXND1,GCNT1,SCD5,PTGDR,CHN1,ADAM8,F2R,YPEL1,ZEB2,SLFN12L,GLCCI1,MXRA7,ADCY9,B4GALT5,MAP3K5,LRP8,PYHIN1,PRDM1,EOMES,STK3,BMPR1A,ADRB2,ANTXR2,GBP5,PTPRM,CLIC1,SV2A,AHNAK,NBEAL2,GLUL,OSBPL7,JOSD1,TPRG1,CCR3,CCL5,KRTAP5-2,DNAJC1,GFPT2,KLRB1,RAB27A,KPNA2,NPC1,CACNB3,NIBAN1,WIPI1,RNF166,WEE1,RNF19A,ST7,ANXA2P2,FOSL2,PARP8,ANXA5,DUSP2,PIK3R3,TBX21,RASGEF1A,CLDND1,RDX,NR4A3,CST7,SH2D2A,ST8SIA1,NAA50,ARHGAP35,NCAPH,SOX13,RGS2,MXD4,LGALS1,TGFB1,ZFYVE28,TGFBI,S1PR5,SLC6A6,TGFBR3,LIMK1,THBS1,CMIP,FADS3,LRIG1,TARP,OSBPL3,GZMA,GZMK,HEATR9,MYO1F,AUTS2,CD58,CD68,PAM,RORA,FAM53B,DUSP10,CTNNA1,SLC4A4,SLAMF7,CLCF1,PEA15,STOM,MYBL1,UEVLD,MAF
**TNvsTEM_UP**	(*18*)	FAM153CP,ANKRD31,KRT72,RGMB,KANSL1L,ZNF135,NOG,ZNF658,HLA-DOA,TSHZ2,PDK1,GJB6,SCOC-AS1,GATM,METAP1D,BCL9,CHKA,CHML,PRAG1,TESPA1,PTK2,KRT73,CHMP7,CEP68,NELL2,SULT1B1,ADPRM,STXBP1,UBE2E2,EDAR,PTPRK,ABRAXAS1,CDNF,GNAI1,TAF4B,CAMSAP2,ZBTB18,TARBP1,TMEM220,ADGRA3,SELL,USP53,PITPNM2,TPST1,RBM11,SDK2,SGTB,DSC1,PIM2,NRCAM,TIMD4,LRRN1,BEND5,BZW2,PPFIBP2,CARMIL1,WNT7A,ARHGEF18,GAL3ST4,NBEA,FAM117B,OCM2,LDHB,SLC40A1,GIPC3,ZNF563,CCNE1,SLC7A8,MICU3,FAM153B,SUGCT,LMO7,ZNF540,PRKAR1B,OLFM2,DEPDC7,CCDC102B,LEF1,ZNF542P,DCBLD2,IL6ST,MYB,RASGRP2
**TSCMvsTEM_DOWN**	(*18*)	EOMES,NPC1,PIK3AP1,CACNB3,PPP2R2B,THBS1,APOBEC3D,KLRF1,MAN1A1,ADRB2,AGO4,ERN1,PTPRM,CLIC1,ARHGAP10,ANXA2P2,CYTH3,NBEAL2,MATK,SYTL2,TBC1D2B,MYO1F,AUTS2,CD58,APOBEC3G,TPRG1,PLEKHA5,GNLY,DUSP10,GNAO1,SH2D2A,PLXND1,NAA50,GFPT2,SLC4A4,PTGDR,DTHD1,ADAM8,GLCCI1,SLFN12L,MXRA7,RAB27B,MAF
**TSCMvsTEM_UP**	(*18*)	CCNE1,SELP,SELL,CEP68,HAVCR1,CRTC3,FAM153B,OCM,ADPRM,NOG,DSC1,TIMD4,LMO7,BZW2,BEND5,PPFIBP2,CARMIL1,WNT7A,ZC3H12D,SESN3,PELI1,FAM117B,IGSF9B,OCM2,METAP1D,TAF4B,ZSWIM1,LEF1,LDHB,ZBTB18,MYB
**TSCMvsTN_DOWN**	(*18*)	EDAR,NRCAM,LRRN1,PTPRK,CYP2J2,OSBPL5,CAMSAP2,MEST,KANSL1L,SLC40A1,NBEA,RBM11,OCRL,IL6ST,SDK2,IGF1R,DEPDC7,KCNQ5
**TSCMvsTN_UP**	(*18*)	EOMES,HAVCR1,NIBAN1,THBS1,GPR15,TSPAN18,ANK3,TIGIT,LRIG1,PTPRM,NETO2,CLIC1,WEE1,SNORD115-11,MICAL2,ANXA2P2,AHNAK,FOSL2,EDARADD,TBX21,ENTPD1,GZMK,MYO1F,ARID5B,CD58,TENT5C,GPRIN3,CLDND1,JOSD1,IRF4,CCL5,CST7,IGSF9B,USP46,GPR183,ST8SIA1,PLXND1,FAS,NCAPH,F2R,ITGB1,IL2RB,SLAMF1,ZEB2,KLRD1,ST6GALNAC2,YARS1,MAP3K1,MYBL1,STOM,SLC27A2,EPHA4,MCOLN2
**Up_Treg_vs_Down_Teff_0h**	REACTOME	NUSAP1,CDKN2A,HDAC9,RASGRP4,PTTG1,IL32,PDE4A,LAYN,TOX,TP53INP1,SWAP70,HLA-DMA,HLA-DMB,PCLAF,HLA-DPA1,HLA-DPB1,HLA-DPB2,ACOT9,HLA-DQB2,HLA-DRA,HLA-DRB1,GJB6,HLA-DRB4,CEACAM4,BARD1,TENT5C,GPR55,ACTN4,NCF4,TSHR,SEMA3G,RRM2,CEP128,ITGB1,FRMD4B,FGL2,PYHIN1,PRDM1,TYMS,S100A4,GDPD5,ADPRH,RTKN2,PTTG3P,NTNG2,FCRL1,FCRL3,SGMS1,UTS2,FCER1G,GBP5,ST8SIA6,CIITA,SYT11,HPGD,GLRX,MKI67,SYT4,NINJ2,CCR3,CCR4,CCR5,CCR6,VAV3,JAKMIP1,BFSP2,SKAP2,METTL7A,ITPRIPL1,PMAIP1,PMCH,FOXP3,SELP,CARD17,SELPLG,CCR10,NIBAN1,IKZF2,MIR4435-

		2HG,SLC2A8,PI16,WEE1,C12orf75,LMCD1,ANXA2,ANXA2P1,ANXA2P2,ANXA2P3,ANXA5,ICA1,DUSP4,CNTNAP1,LAIR2,ARHGEF12,SHMT2,CASP8,OAS1,CLDND1,E2F2,BCAS1,STAM,NABP1,RAB11FIP1,FAS,FAM160B1,NCAPH,ZNF365,RGS1,SLC1A4,LGALS1,LGALS3,IKZF4,CEP55,FANK1,SPATS2L,ZC2HC1A,TIGIT,SLC14A1,CDCA7,TK1,MYO1F,CD58,TNFRSF13B,DUSP10,SETD7,PRR11,ATP1B1,LINC02694,TRIB1,HACD1,MIAT,MYBL2
**WINTER_HYPOXIA_METAGENE**	(*21*)	CDKN1A,CDKN1B,BHLHE41,HDAC9,EPO,HGF,VPS11,HIF1A,HK1,HK2,RPL36A,PDGFB,GAPDH,CENPX,ACAT1,SPP1,MAP4,PDK3,EIF4A3,HLA-DQB1,PSMA3,INSIG1,HSPH1,ETS1,GBE1,NOS2P2,RIOK3,IRF6,PSMD9,SIRPA,BCL2L1,PFKFB1,BCL2L2,PFKFB3,PFKFB4,HMOX2,NR4A1,HMOX1,ITGA5,PFKL,PGAM1,F3,PGF,NOS2P1,PTGS1,PTGS2,PGK1,PGK2,DDIT3,MXI1,PGM1,AK3,TCEAL1,ABCB1,FABP5,PLIN2,BIK,TXN,CITED2,ADM,QSOX1,ADORA2B,MET,JUN,LONP1,BNIP3,BNIP3L,NDRG1,SAT1,BPI,CLK1,PIM1,CXCR4,CD99,NFIL3,SGSM2,NFKB1,NFKB2,BTG1,GLRX,MIF,CNOT7,PKM,SLC6A10P,TIMM17A,FGF3,DKC1,AK2,UMPS,KDR,PLAU,PLAUR,HERC3,BATF3,ALDH1A1,MMP2,MMP7,ALDH1A3,BACE2,ALDOA,MMP13,HILPDA,ALDOC,PLOD2,NOS1,NOS2,HSPA5,NOS3,FKBP4,PNP,VEGFA,VEGFB,VEGFC,HSPD1,CA9,CA12,MPI,GPI,VIM,COL4A5,COL5A1,COL5A2,DDIT4,P4HA2,FLT1,DR1,KRT14,HYOU1,ANGPT2,SRSF6,PIM2,FN1,CALD1,KRT18,PNN,ZNRD2,KRT19,HAP1,POLM,NT5E,SLC6A16,ANXA1,ANXA2,TBPL1,FOS,ANXA5,ICAM1,ID2,BIRC2,CP,COL5A3,SIAH2,HBP1,RELA,SIN3A,ODC1,PPARA,NR3C1,GPRC5A,PPAT,TEK,LDHA,IFI27,LDHB,SYT7,TF,BHLHE40,PAICS,LEP,SLC2A1,EDN1,EDN2,LGALS1,SLC2A3,SLC3A2,TFF3,TFRC,TGFA,TGFB1,TGFB3,TXNIP,TGFBI,CCNG2,SLC6A8,SLC6A6,ARHGAP5,CCT6A,TGM2,TH,THBS1,THBS2,MT1L,EFNA1,ELL2,IGF2,RNASEL,IGFBP1,IGFBP2,EGF,ARSL,IGFBP3,ART1,IGFBP5,STC2,SLC16A2,SLC16A1,P4HA1,EGR1,SLC20A1,LOX,RRP9,MTL3P,NSG1,ANGPTL4,RBPJ,SERPINE1,ERRFI1,SERPINB2,LRP1,ELF3,FTL,PROK1,CDK1,TNFAIP3,CTSD,ENG,MUC1,ENO1,ENO3,ENPEP,HERPUD1,IL6,XRCC6,TP53,CXCL8,HDAC1,TPBG,TPD52,SORL1,TPI1
**WNT_StefaniSpranger**	(*22*)	MYC,HNF1A,APC2,TCF12,EFNB3,VEGFA

Reference:

1. M. Carleton *et al.*, Pharmacodynamics (PD) and genomic profiling of pts treated with cabiralizumab (cabira) plus nivolumab (NIVO) provide evidence of on-target tumor immune modulations and support future clinical applications. *J Clin Oncol* **36**, (2018).
2. L. Jerby-Arnon *et al.*, A Cancer Cell Program Promotes T Cell Exclusion and Resistance to Checkpoint Blockade. *Cell* **175**, 984-997 e924 (2018).
3. M. S. Rooney, S. A. Shukla, C. J. Wu, G. Getz, N. Hacohen, Molecular and genetic properties of tumors associated with local immune cytolytic activity. *Cell* **160**, 48-61 (2015).
4. S. Spranger, D. Dai, B. Horton, T. F. Gajewski, Tumor-Residing Batf3 Dendritic Cells Are Required for Effector T Cell Trafficking and Adoptive T Cell Therapy. *Cancer Cell* **31**, 711-723 e714 (2017).
5. E. J. Wherry *et al.*, Molecular signature of CD8+ T cell exhaustion during chronic viral infection. *Immunity* **27**, 670-684 (2007).
6. C. X. Dominguez *et al.*, Single-Cell RNA Sequencing Reveals Stromal Evolution into LRRC15 Myofibroblasts as a Determinant of Patient Response to Cancer Immunotherapy. *Cancer Discov* **10**, 232-253 (2020).
7. C. J. Luckey *et al.*, Memory T and memory B cells share a transcriptional program of self-renewal with long-term hematopoietic stem cells. *Proc Natl Acad Sci U S A* **103**, 3304-3309 (2006).
8. A. L. Harris, Hypoxia--a key regulatory factor in tumour growth. *Nat Rev Cancer* **2**, 38-47 (2002).
9. W. Chen *et al.*, Targeting renal cell carcinoma with a HIF-2 antagonist. *Nature* **539**, 112-117 (2016).
10. M. Ayers *et al.*, IFN-gamma-related mRNA profile predicts clinical response to PD-1 blockade. *J Clin Invest* **127**, 2930-2940 (2017).

11. R. Dummer *et al.*, Combined PD-1, BRAF and MEK inhibition in advanced BRAF-mutant melanoma: safety run-in and biomarker cohorts of COMBI-i. *Nat Med* **26**, 1557-1563 (2020).
12. K. Yoshihara *et al.*, Inferring tumour purity and stromal and immune cell admixture from expression data. *Nat Commun* **4**, 2612 (2013).
13. W. Hugo *et al.*, Genomic and Transcriptomic Features of Response to Anti-PD-1 Therapy in Metastatic Melanoma. *Cell* **165**, 35-44 (2016).
14. H. Liu *et al.*, Tumor-derived IFN triggers chronic pathway agonism and sensitivity to ADAR loss. *Nat Med* **25**, 95-102 (2019).
15. M. C. Wagle *et al.*, A transcriptional MAPK Pathway Activity Score (MPAS) is a clinically relevant biomarker in multiple cancer types. *Npj Precis Oncol* **2**, (2018).
16. Y. Y. Natsuki Kawana, Yoshihiro Kino, Jun-ichi Satoh, Molecular Network of NLRP3 Inflammasome Activation-Responsive Genes in a Human Monocyte Cell Line. *Austin Journal of Clinical Immunology* **1**, 1017 (2014).
17. S. Mariathasan *et al.*, TGFbeta attenuates tumour response to PD-L1 blockade by contributing to exclusion of T cells. *Nature* **554**, 544-548 (2018).
18. L. Gattinoni *et al.*, A human memory T cell subset with stem cell-like properties. *Nat Med* **17**, 1290-1297 (2011).
19. R. Cristescu *et al.*, Pan-tumor genomic biomarkers for PD-1 checkpoint blockade-based immunotherapy. *Science* **362**, (2018).
20. C. Sautes-Fridman, F. Petitprez, J. Calderaro, W. H. Fridman, Tertiary lymphoid structures in the era of cancer immunotherapy. *Nat Rev Cancer* **19**, 307-325 (2019).
21. S. C. Winter *et al.*, Relation of a hypoxia metagene derived from head and neck cancer to prognosis of multiple cancers. *Cancer Res* **67**, 3441-3449 (2007).
22. S. Spranger, R. Bao, T. F. Gajewski, Melanoma-intrinsic beta-catenin signalling prevents anti-tumour immunity. *Nature* **523**, 231-235 (2015).
