# Supplementary material for: Defining Non–small Cell Lung Cancer Tumor Microenvironment Changes at Primary and Acquired Immune Checkpoint Inhibitor Resistance Using Clinical and Real-World Data
Source: Cancer Res Commun. 2025 Jun 30;5(6):1049–59. doi: 10.1158/2767-9764.CRC-24-0605 (PMC12207206; doi:10.1158/2767-9764.CRC-24-0605)
Supplement: Supplementary Table S2 — Patient characteristics of three Tempus cohorts for age, gender, race and PD-L1 class [file crc-24-0605_supplementary_table_s2_suppst2.pdf]

Supplementary Table S2. Patient characteristics of three Tempus cohorts for age, gender, race and PD-L1 class.

	Cohort 1, N = 415	Cohort 2, N = 326	Cohort 3, N = 56
**Age at Diagnosis**			
Median (Q1, Q3)	67 (61, 73)	63 (56, 70)	61 (54.75, 69)
**Gender**			
Female	220 (53%)	151 (46.3%)	34 (60.7%)
Male	195 (47%)	175 (53.7%)	22 (39.3%)
**Race**			
White	256 (61.7%)	186 (57.1%)	35 (62.5%)
Black or African American	44 (10.6%)	34 (10.4%)	6 (10.7%)
Asian	10 (2.4%)	4 (1.2%)	2 (3.6%)
Other Race	23 (5.5%)	6 (5.5%)	1 (1.8%)
Unknown	81 (19.5%)	94 (28.8%)	12 (21.4%)
**PD-L1 class**			
< 1%	106 (25.5%)	171 (52.4%)	15
1 – 49%	77 (18.6%)	73 (22.4%)	5
> 50%	227 (54.7%)	81 (24.8%)	4
